# Human antibodies neutralizing diphtheria toxin *in vitro* and *in vivo*

**DOI:** 10.1038/s41598-019-57103-5

**Published:** 2020-01-17

**Authors:** Esther Veronika Wenzel, Margarita Bosnak, Robert Tierney, Maren Schubert, Jeffrey Brown, Stefan Dübel, Androulla Efstratiou, Dorothea Sesardic, Paul Stickings, Michael Hust

**Affiliations:** 1 Technische Universität Braunschweig, Institute for Biochemistry, Biotechnology and Bioinformatics, Department of Biotechnology, Braunschweig, Germany; 2 National Institute for Biological Standards and Control (NIBSC), Division of Bacteriology, Potters Bar, United Kingdom; 3 PETA International Science Consortium Ltd, London, United Kingdom; 4 WHO Collaborating Centre for Diphtheria and Streptococcal Infections, London, UK

**Keywords:** Antibody therapy, Applied immunology

## Abstract

Diphtheria is an infectious disease caused by *Corynebacterium diphtheriae*. The bacterium primarily infects the throat and upper airways and the produced diphtheria toxin (DT), which binds to the elongation factor 2 and blocks protein synthesis, can spread through the bloodstream and affect organs, such as the heart and kidneys. For more than 125 years, the therapy against diphtheria has been based on polyclonal horse sera directed against DT (diphtheria antitoxin; DAT). Animal sera have many disadvantages including serum sickness, batch-to-batch variation in quality and the use of animals for production. In this work, 400 human recombinant antibodies were generated against DT from two different phage display panning strategies using a human immune library. A panning in microtiter plates resulted in 22 unique *in vitro* neutralizing antibodies and a panning in solution combined with a functional neutralization screening resulted in 268 *in vitro* neutralizing antibodies. 61 unique antibodies were further characterized as scFv-Fc with 35 produced as fully human IgG1. The best *in vitro* neutralizing antibody showed an estimated relative potency of 454 IU/mg and minimal effective dose 50% (MED50%) of 3.0 pM at a constant amount of DT (4x minimal cytopathic dose) in the IgG format. The targeted domains of the 35 antibodies were analyzed by immunoblot and by epitope mapping using phage display. All three DT domains (enzymatic domain, translocation domain and receptor binding domain) are targets for neutralizing antibodies. When toxin neutralization assays were performed at higher toxin dose levels, the neutralizing capacity of individual antibodies was markedly reduced but this was largely compensated for by using two or more antibodies in combination, resulting in a potency of 79.4 IU/mg in the *in vivo* intradermal challenge assay. These recombinant antibody combinations are candidates for further clinical and regulatory development to replace equine DAT.

## Introduction

**Figure 1:**
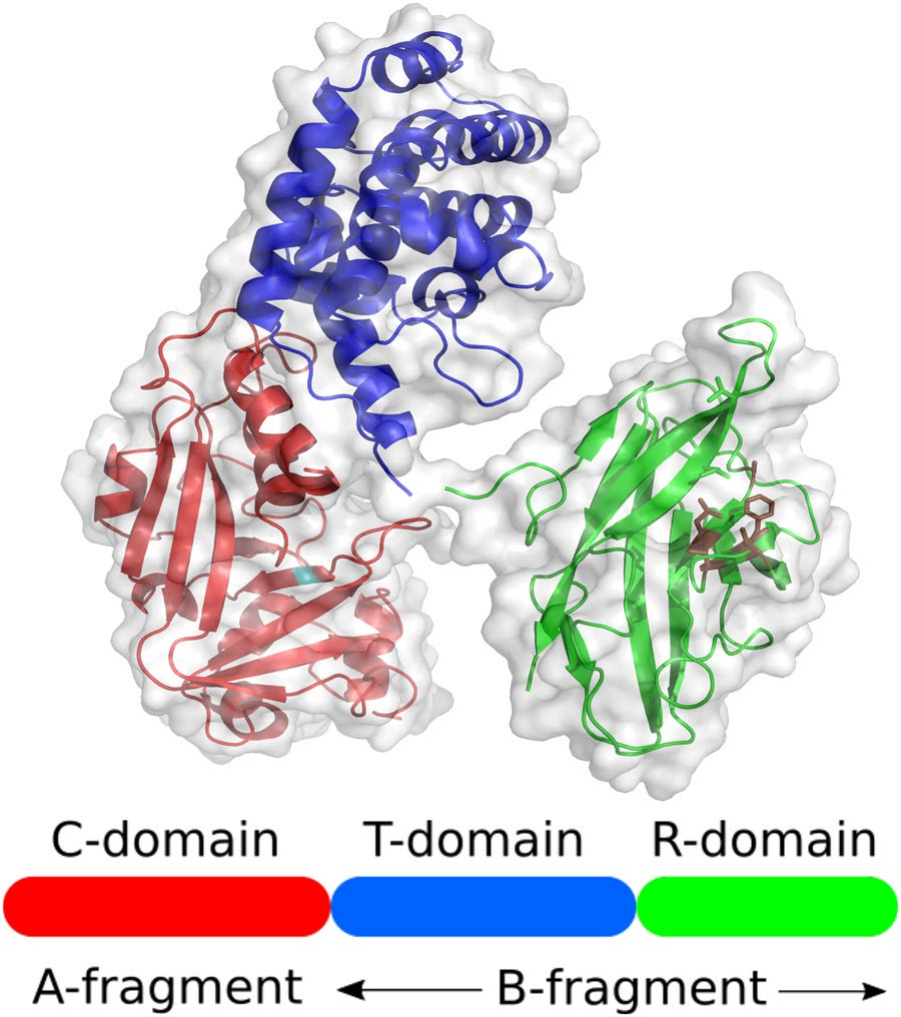
Protein structure of diphtheria toxin: C-domain (red), T-domain (blue), R-domain (green) (modified from PhD thesis^84^). A) crystal structure, the catalytic center is marked in cyan and the amino acids binding the HB-EGF receptor are marked brown (modified from pdb 1ddt^85^). B) Schematic structure of DT.

Diphtheria is an infectious disease and caused by *Corynebacterium diphtheriae*. The bacterium primarily infects the throat and upper airways but can also affect other body sites. The produced toxin can spread through the bloodstream and affect organs, such as the heart and kidneys. In severe cases, diphtheria toxin (DT) may cause myocarditis or peripheral neuropathy. Due to a membrane of dead tissue over the throat and tonsils, swallowing and breathing can be difficult. The disease is spread through direct physical contact or by coughing or sneezing of infected individuals^1–3^. Diphtheria is fatal in 5–10% of cases, but children under the age of five have a mortality rate of up to 20%. Treatment involves antibiotics to kill the bacteria (erythromycin or penicillin for 14 days) and administering of diphtheria antitoxin (DAT) to neutralize the effects of the toxin^4–6^. *C. diphtheriae* was identified as the causative agent of diphtheria in 1883 and in 1888 the diphtheria toxin was first described in the culture medium of *C. diphtheriae*^7^. The gene for DT is encoded on a corynebacteriophage^8,9^ and the toxin is secreted as a single polypeptide chain of 535 amino acid residues with a molecular weight of 58 kDa^10,11^. Mild trypsinization and reduction of DT *in vitro* generates two fragments, A and B, which remain covalently attached by an inter-chain disulfide bond^12^. Fragment A contains the enzymatic activity, whereas fragment B binds the protein to the cell surface receptors and promotes translocation of fragment A to the cytoplasm^13,14^. DT is composed of three structural domains: the catalytic domain (C) corresponds to fragment A (21 kDa) while the transmembrane (T) domain (20 kDa) and receptor binding (R) domain (17 kDa) comprise fragment B (37 kDa) (Fig. 1)^15^. Diphtheria toxin binds with the receptor binding domain to heparin-binding epidermal growth factor (HB-EGF) on the cell surface of susceptible cells. This binding triggers receptor-mediated endocytosis of the toxin and the acidic conditions in the endosome lead to a conformational change and the translocation domain of DT to form a channel through the endosomal membrane to transport the C-domain to the cytosol^16–18^. In the cytosol, the C-domain catalyzes the NAD+ -dependent ADP-ribosylation of elongation factor 2 (EF-2) of the ribosome resulting in an inactivation of the protein synthesis and cell death by apoptosis^19,20^.

In 1890, Emil von Behring and Shibasaburō Kitasato found that the serum of immunized animals is protective against DT^21^. This serum therapy was a breakthrough for the treatment of diphtheria, especially for children, and was awarded with the first Nobel Prize for medicine in 1901. Despite the introduction of effective vaccination programmes against diphtheria, gaps in immunization coverage still exist, meaning that diphtheria remains endemic in some areas. Even in populations with good immunization coverage, isolated cases still occur. In recent years, significant diphtheria outbreaks have occurred in countries or regions with a collapsed health system because of political instability or civil war, e.g. Yemen, Venezuela or the Rohingya refugee camps in Cox’s Bazar (Bangladesh), resulting in failed vaccinations of children^22–25^. In all clinical cases, the primary therapeutic option is still treatment with diphtheria antitoxin (DAT) produced by hyper-immunization of horses.

Production of therapeutic antibodies in horses raises ethical issues surrounding the use of animals, especially by substandard housing and veterinary care of the horses, and there are strict requirements for ensuring freedom from adventitious agents. Equine hyperimmune sera contain a large and varied amount of different antibodies with unknown specificity and, because of the nature of the product, there is potential for variations in quality between different batches. The human immune system may develop antibodies against foreign antigens introduced from administration of the animal sera, which leads to the formation of immune complexes, which can precipitate in joints or small vessels, activating the complement cascade and initiating a systematic and potentially serious inflammatory response, a condition known as serum sickness^26–28^. Today, DAT is in scarce supply and frequently unavailable to patients because of discontinued production in several countries^29^. There is an urgent need for an alternative to replace the equine DAT, therefore, new treatment options with recombinant fully human antibodies are desirable. Recombinant human antibodies are sequence defined, produced in cell culture and as they are human proteins, serum sickness can be avoided. These advantages of recombinant antibodies make them ideal therapeutics against pathogens and toxins^30,31^.

The most common technology to generate recombinant human therapeutic antibodies is antibody phage display^32^. Antibody phage display allows the selection of antibody fragments, mainly single chain fragment variable (scFv) or fragment antigen binding (Fab), directly from human antibody gene libraries *in vitro*. In brief, antibody fragments are displayed on the surface of filamentous M13 phage particles by linking the antibody to the phage protein III. The corresponding antibody gene fragment is encoded on a phagemid packaged in the phage particles so that, phenotype and genotype are coupled. In a selection process called “panning”, antibody phage particles are incubated with the antigen, non-binding phage particles are washed away and the binding antibody phage particles are eluted. This process is repeated 2–3 times. Subsequently, monoclonal antibodies are produced and screened for antigen binding, e.g. ELISA on antigen, or in functional assays, e.g. neutralization assays^33–36^. Because the gene encoding the antibody fragment is directly accessible, the antibodies can be converted into other antibody formats such as scFv-Fc or IgG, for further experiments and applications^37,38^. Antibody phage display is a well validated tool to generate recombinant antibodies against toxins for diagnostic and therapeutic purposes^39–44^. An overview of antibodies generated by phage display against pathogens and toxins is given by Kuhn *et al*.^31^. An overview on phage display derived therapeutic antibodies is given by Frenzel *et al*.^32^.

In this work, recombinant human antibodies against DT were generated using phage display to neutralize DT *in vitro* and *in vivo* and to identify potential future alternatives to equine DAT as a frontline therapy for diphtheria.

## Results

### Immune antibody library construction

Three individuals received a regular booster immunization with an adsorbed diphtheria and tetanus vaccine. Seven days after immunization, EDTA-treated blood samples were collected and PBMCs extracted with Ficoll. An average concentration of 4.7 × 10^7^ PBMC/mL was counted. Two immune antibody gene libraries were constructed. For the first library, the total PBMCs were used and the RNA was isolated resulting in 10.5 µg total RNA used for cDNA synthesis. The cDNAs derived from the blood of each person was kept separated in the construction process of the total PBMC library and the final sublibraries were combined. This first immune library was called VJN library. For the second library, the freshly prepared PBMCs were stained with a florescence conjugated CD138^+^ antibody to detect the antibody-producing B cells. In the next step, the stained cells were selected by fluorescence activated cell sorting (FACS). 8.8 × 10^7^ PBMCs were sorted, resulting in the isolation of 2,501 CD138^+^ cells ( = 0.003%). Their RNA was extracted (350 ng total RNA) and transcribed in cDNA. This second immune library was called the CD138+ library.

The cDNA from total PBMC and CD138^+^ sorted cells was used to amplify the gene sequences of the variable regions of immunoglobulin heavy and light chains (VH and VL). The VH and VL were cloned into the phage display vector pHAL51. Separate lambda and lappa libraries were constructed. The maximal diversity of the sub-libraries, as determined by the number of independent clones, was between 1.3–3.4 × 10^8^, with an insert rate of 73–95%. Subsequently, the libraries were packaged with Hyperphage^45,46^ to display the scFv antibody coupled to the pIII on the phage. For packaging, the sub-libraries of the total PBMC approach were pooled resulting in the VJN library. The lambda and kappa libraries were still kept separate. This resulted in phage titers of 7.4 × 10^12^ cfu/mL for VJN lambda library, 7.2 × 10^12^ cfu/mL for VJN kappa library, 2.6 × 10^12^ cfu/mL for CD138+ lambda library and 6.0 × 10^12^ cfu/mL for CD138+ kappa library.

### Selection and screening of anti-diphtheria toxin antibodies

For antibody selection, both VJN and CD138+ immune libraries were used. Two different panning and screening strategies were chosen for antibody selection. In the first approach, the antigen was immobilized on a microtiter plate (MTP) and the screening was performed by ELISA of secreted soluble scFv fragments to determine antigen binding. In the second strategy, a panning in solution was performed using biotinylated antigen followed by a functional screening of DT neutralization using the Vero cell assay. Using both approaches, 400 antibodies binding to DAT were selected in total. The overview showing all panning strategies and the subsequent steps is given in Table 1.

**Table 1:** Overview of all selected antibodies separated by library type, light chain type, panning strategy, screening strategy and further production and characterization steps. *Three of the 15 antibodies were produced in two variants: (1) original phage display selected sequence and (2) modified framework 1 sequence according to the corresponding V-gene germline sequence. This data was generated in a PhD thesis^84^.

Library type	Light chain type	Panning strategy	Screening strategy	Antibody selection campaign	Selected mabs	Produced & purified as scFv-Fc	Binding in ELISA as scFv-Fc	Neutra-lizing as scFv-Fc	Produced & purified as IgG	Binding in ELISA as IgG	Neutra-lizing as IgG
Immune	Lambda+ Kappa (mix)	MTP	scFv binding	ewe191	67	51/67	35/51	10/35	10/10	10/10	9/10
Immune (CD138+ selected)	Lambda+ Kappa (mix)	MTP	scFv binding	ewe192	65	50/65	38/50	12/38	12/12	12/12	12/12
Immune	Lambda	in solution	scFv-Fc neutralization	ewe371	71	13/71	13/13	13/13	1/13	1/1	1/1
Immune	Kappa	in solution	scFv-Fc neutralization	ewe372	66	15/66	18*/15	18/18	10/15	10/10	10/10
Immune (CD138+ selected)	Lambda	in solution	scFv-Fc neutralization	ewe374	54	3/54	3/3	3/3	0/3	—	—
Immune (CD138+ selected)	Kappa	in solution	scFv-Fc neutralization	ewe375	77	5/77	5/5	5/5	2/5	2/2	2/2
Sum					400	137	112	61	35	35	34

Using the first strategy relying on antigen immobilization on MTPs, 132 scFvs were isolated in total from both immune libraries. For the analysis of the individual antibody sequence, a BstNI digestion and agarose gel electrophoresis was initially performed to exclude duplicate clones which have identical digestion patterns (data not shown), followed by sequencing of the scFv encoding gene inserts. 101 of these scFv were identified as unique after sequencing. One antibody contained a stop codon in the sixth position of the VH chain (ewe192-B5), despite being produced in functional form in *E. coli*. For this antibody, the respective germline sequence (IGHV4-34*01) encodes the amino acid glutamine (Q) at position 6. After polymerase incomplete primer extension (PIPE) cloning, the stop codon was changed to the amino acid Q. This modified antibody was labelled as ewe192-B5(P). For all further characterizations, the scFv antibodies against DT were converted to an IgG-like scFv-Fc format. The genes encoding scFv antibody fragments were cloned into the pCSE2.7-hIgG1-Fc expression vector and produced as scFv-Fc.

In the second strategy using panning in solution, a pull down was performed using magnetic streptavidin beads to capture the antibody phage bound to the biotinylated DT in solution. The successful enrichment of DT-binding phage by panning in solution was followed by a phage ELISA (data not shown). The antibody pool was directly cloned into the vector pCSE2.7-hIgG1-Fc-XP for production as scFv-Fc fusions. 384 monoclonal scFv-Fc were selected for the screening assay. All antibodies were produced in EXPI293F cells in 96 deep well plates. The produced antibodies were secreted into the growth medium and the supernatant was used for performing the neutralization assay in Vero cells. A four-point serial dilution of the supernatant was incubated with a constant amount of diphtheria toxin (4 × minimal cytopathic dose, 4 × MCD) and, after 6 days of incubation with Vero cells, the cytopathic effect was measured with an Thiazolyl Blue Tetrazolium Bromide (MTT) assay. These produced antibodies were analyzed regarding their neutralization efficacy using the Vero cell TNT assay^47^ (data not shown). In total, 268 scFv-Fc showed neutralization. The 36 antibodies showing the best neutralization were selected for further analysis.

Three antibodies (ewe372-C10, ewe372-D3 and ewe372-H1) contained a stop codon in the sixth position of the VH chain. For these three antibodies, the corresponding germline gene (IGHV4-34) encodes the amino acid glutamine (Q) at position six. A point mutation was introduced to change the stop codon to the amino acid Q. These three antibodies had the amino acid Serin (S) at position 7 of VH, but according to the corresponding germ line gene, the amino acid tryptophan (W) should occur at this position. Hence, two versions were developed for each of the three antibodies and named after their seventh amino acid position ewe372-C10-S/ewe372-C10-W, ewe372-D3-S/ewe372-D3-W and ewe372-H1-S/ewe372-H1-W. After these modifications, a total of 39 scFv-Fc antibodies from the panning in solution were further analyzed.

### *In vitro* neutralization in scFv-Fc format

**Figure 2:**
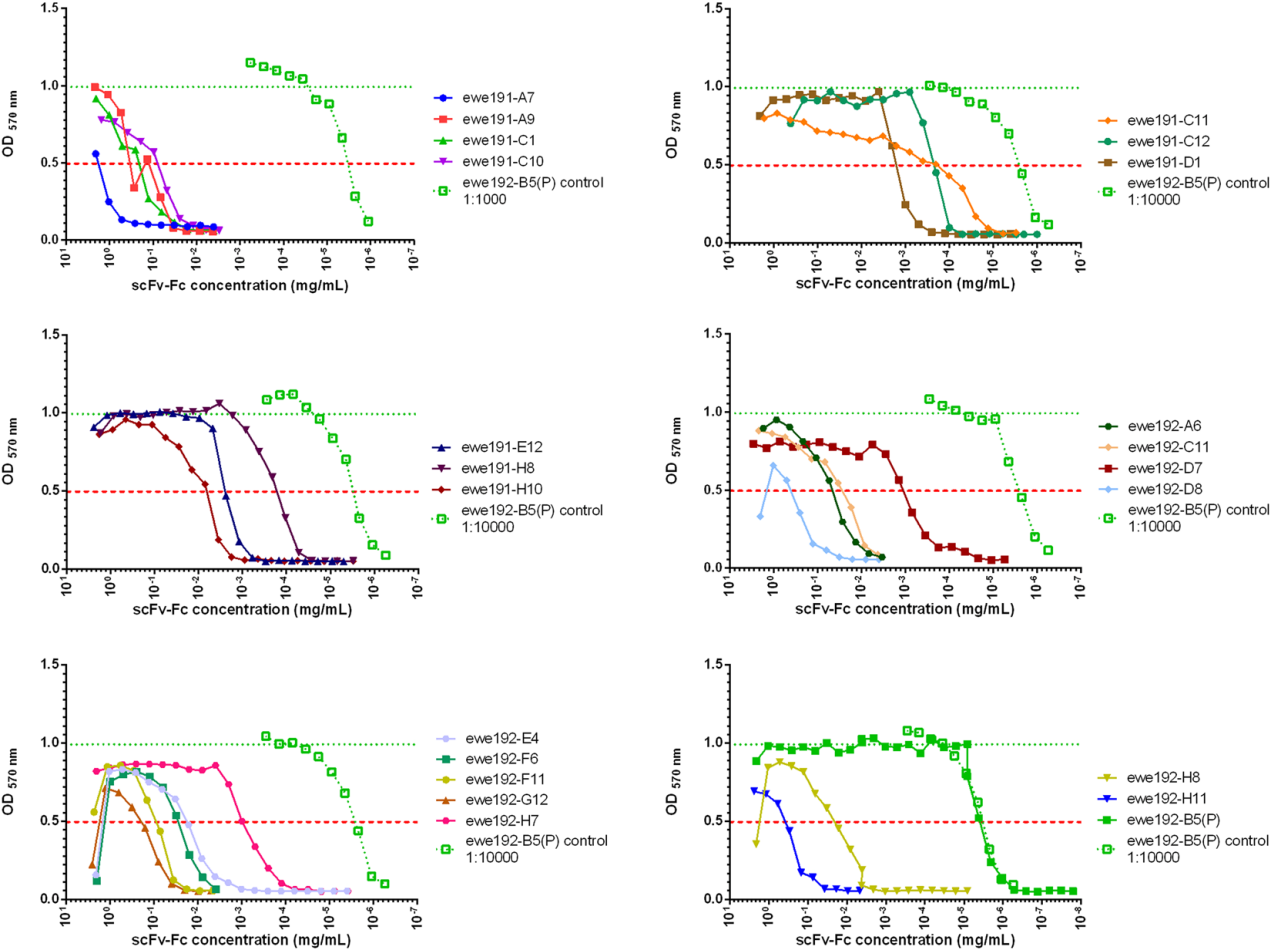
Cell based neutralization assay (using a DT dose level of 4 × MCD) for 21 scFv-Fc derived from the panning in MTPs. The OD for untreated (control) Vero cells is shown as a horizontal green dotted line. The threshold for neutralization (defined as 50% of the control cell OD) is shown as a horizontal red dotted line. The antibody ewe192-B5(P) (green, open symbol and dotted line) was used as positive control and to standardize the assays performed in parallel on different MTPs. Figure modified from PhD thesis^84^.

**Figure 3:**
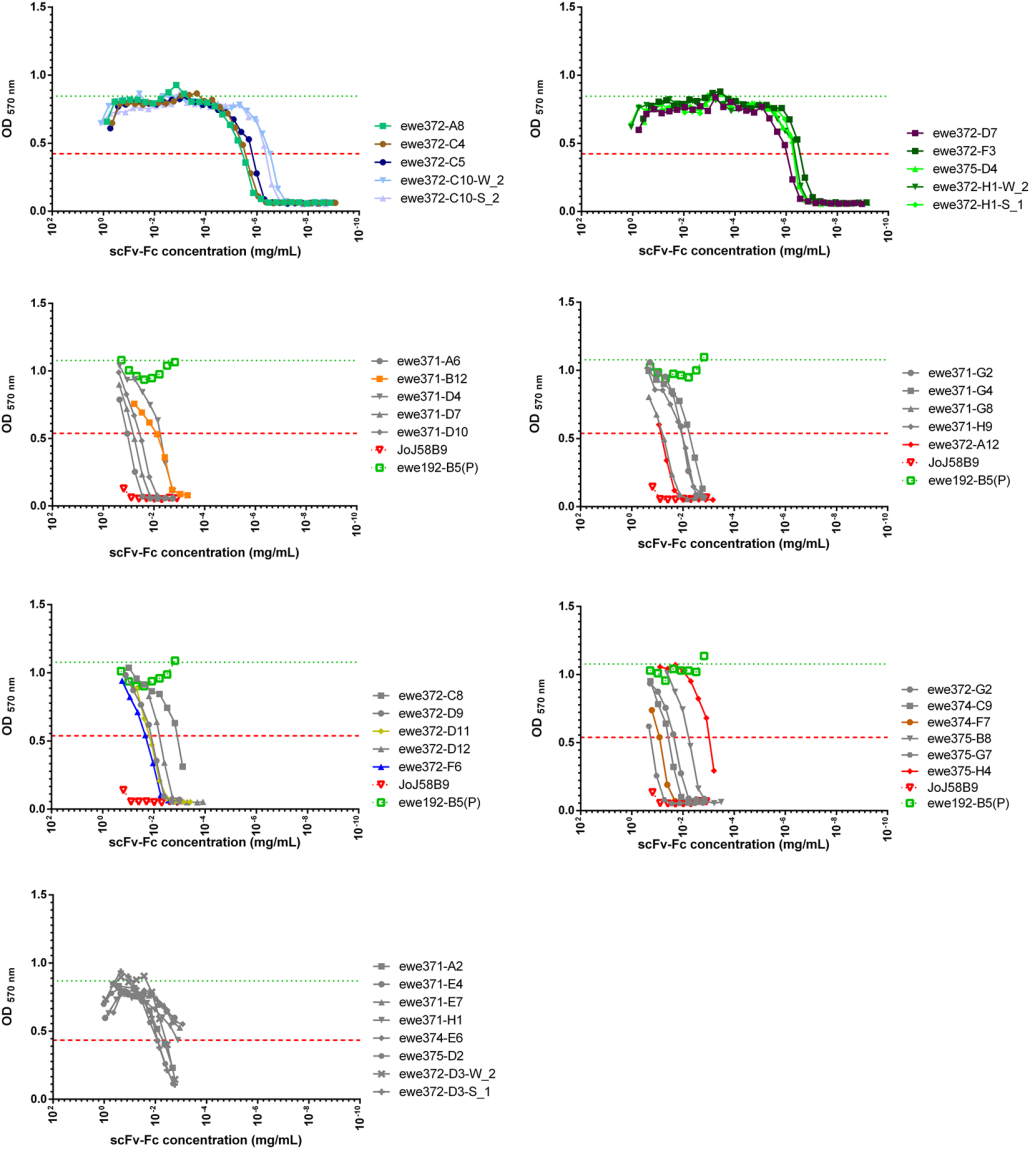
Cell based neutralization assay for 39 scFv-Fc antibodies derived from the panning in solution. Untreated Vero cells are given as green dotted line. The threshold for neutralization is given as red dotted line (50% of untreated Vero cells). The anti-glucan antibody JoJ58B9 (red, open symbols and dotted line) is the negative control and the antibody ewe192-B5(P) (green, open symbol and dotted line) is the positive control. Antibodies represented in coloured symbols were further analysed and antibodies represented in grey symbols were excluded. Figure modified from PhD thesis^84^.

The *in vitro* neutralization capacity of the 101 unique scFv-Fc derived from the panning in MTPs, and the 39 best neutralizing scFv-Fc derived from the panning in solution, were further analyzed to determine the minimal effective dose 50% (MED50%) (lowest antibody concentration with more than 50% neutralization efficacy) using the Vero cell TNT assay^47^. Here, a serial dilution of purified scFv-Fc was incubated with a constant amount of DT (4 × MCD) and the cytopathic effect was measured with an MTT assay. 22 of the 101 scFv-Fc derived from the panning in MTPs showed DT neutralization (Fig. 2). The neutralization capacity of the scFv-Fc derived from the panning in solution are shown in Fig. 3. The *in vitro* neutralization capacity was determined to be between 3.3 pM (3.7 × 10^−7^ mg/mL) and 18.8 µM (2 mg/mL) at a constant amount of DT (4 × MCD) (Table 2). Most of the scFv-Fc had a neutralization efficacy in the nanomolar range. The antibody germline V-genes of all 61 neutralizing antibodies are given in Table 3.

**Table 2:** Overview of the half maximal binding concentration (EC50) in the scFv-Fc and IgG format on DT and CRM197 in ELISA. The *in vitro* neutralization capacity in the Vero cell TNT assay (using a toxin dose of 4 × MCD) is shown as MED50% (for scFv-Fc and IgG) and IU/mg (for IgG only). n.d. = not done. For the conversion of nM to mg/mL a mean molecular weight of 110 kD for scFv-Fc and 145 kD for IgG was used. This data was generated in a PhD thesis^84^.

#	Name antibody clone	DT binding				*in vitro* neutralization				
		EC50 (µg/mL) scFv-Fc	CRM197 EC50 (µg/mL) scFv-Fc	EC50 (µg/mL) IgG	CRM197 EC50 (µg/mL) IgG	MED50% (nM) scFv-Fc		MED50% (nM) IgG		IU/mg IgG
						(nM)	(mg/mL)	(nM)	(mg/mL)	
1	ewe191-A7	0.0086	0.0108	0.0244	0.0245	18854.3	2.07397	140.3	0.02034	0.0049
2	ewe191-A9	0.0154	2.8240	0.0264	1.4110	1266.1	0.13927	1098.5	0.15928	0.0013
3	ewe191-C1	0.0101	0.0218	0.0381	0.0311	2360.9	0.25969	818.5	0.11868	0.0017
4	ewe191-C10	0.1748	1.5410	0.3541	1.0200	1821.7	0.20038	n.d.	n.d.	—
5	ewe191-C11	0.0105	0.0122	0.0253	0.0312	7.6	0.00083	0.6	8.7E-05	2.2
6	ewe191-C12	0.0254	0.0200	0.0466	0.0468	7.3	0.00080	2.4	0.000348	0.57
7	ewe191-D1	0.0100	0.0125	0.0266	0.0310	36.9	0.00406	31.2	0.00452	0.044
8	ewe191-E12	0.0459	0.0246	0.0297	0.0329	43.2	0.00475	19.2	0.00278	0.071
9	ewe191-H10	0.0335	0.0839	0.0285	0.0332	65.4	0.00719	119.1	0.01727	0.017
10	ewe191-H8	0.0143	0.0251	0.0578	0.0931	3.9	0.00043	1.0	0.000145	1.45
11	ewe192-A6	0.2031	0.6579	0.4095	40.0800	500.8	0.05509	780.7	0.1132	0.0018
12	ewe192-B5(P)	0.0441	0.0587	0.0535	0.0679	0.075	8.25E-06	0.007	1.02E-06	124
13	ewe192-C11	0.0113	0.0227	0.0337	0.0312	313.8	0.03452	116.7	0.01692	0.012
14	ewe192-D7	0.0119	0.0133	0.0333	0.0291	26.4	0.00290	3.4	0.000493	0.41
15	ewe192-D8	3.9280	3.3680	0.3612	2.4640	4667.8	0.51346	949.3	0.13765	0.0015
16	ewe192-E4	0.0920	0.2222	0.0681	30.9200	301.3	0.03314	35.7	0.00518	0.039
17	ewe192-F11	0.49	2.0760	0.2021	31.6400	1312.7	0.1444	1415.9	0.20531	0.0010
18	ewe192-F6	0.1558	0.5040	0.1380	7.5480	287.9	0.03167	357.6	0.05185	0.0038
19	ewe192-G12	0.89	1.4080	0.5226	34.1600	2911.9	0.32031	180.8	0.02622	0.0076
20	ewe192-H11	0.0143	0.1678	0.0263	0.0453	5506.8	0.60575	1351.3	0.19594	0.0010
21	ewe192-H7	0.0138	0.0144	0.0349	0.0245	18.8	0.00207	10.9	0.00158	0.14
22	ewe192-H8	0.1589	0.3111	0.0938	3.5430	303.6	0.0334	254.0	0.03683	0.0054
23	ewe371-A2	0.08299	0.1176	n.d.	n.d.	77.272	0.0085	n.d.	n.d.	n.d.
24	ewe371-A6	0.03316	0.04078	n.d.	n.d.	20.324	0.002236	n.d.	n.d.	n.d.
25	ewe371-B12	0.01456	0.0141	0.0182	0.0214	1.407	0.000155	1.2028	0.000174	1.15
26	ewe371-D10	0.02058	0.02136	n.d.	n.d.	5.496	0.000605	n.d.	n.d.	n.d.
27	ewe371-D4	0.02356	0.02439	n.d.	n.d.	0.642	7.06E-05	n.d.	n.d.	n.d.
28	ewe371-D7	0.02815	0.03871	n.d.	n.d.	10.621	0.001169	n.d.	n.d.	n.d.
29	ewe371-E4	0.03578	0.1132	n.d.	n.d.	16.373	0.0018	n.d.	n.d.	n.d.
30	ewe371-E7	0.02544	0.03123	n.d.	n.d.	10.134	0.001114	n.d.	n.d.	n.d.
31	ewe371-G2	0.02945	0.04119	n.d.	n.d.	1.107	0.000121	n.d.	n.d.	n.d.
32	ewe371-G4	0.2942	4.738	n.d.	n.d.	0.622	6.842E-05	n.d.	n.d.	n.d.
33	ewe371-G8	0.03405	0.04117	n.d.	n.d.	9.841	0.001082	n.d.	n.d.	n.d.
34	ewe371-H1	0.02254	0.06175	n.d.	n.d.	24.006	0.00264	n.d.	n.d.	n.d.
35	ewe371-H9	0.03138	0.04574	n.d.	n.d.	1.419	0.000156	n.d.	n.d.	n.d.
36	ewe372-A12	0.02153	0.03627	0.0210	0.0291	8.032	0.000883	4.1944	0.000608	0.33
37	ewe372-A8	0.02257	0.02301	0.0239	0.0279	0.0512	5.63E-06	0.0826	1.2E-05	16.6
38	ewe372-C10-S	0.05643	0.2061	n.d.	n.d.	0.00483	5.31E-07	n.d.	n.d.	n.d.
39	ewe372-C10-W	0.04717	0.1936	0.0356	0.0548	0.00551	6.06E-07	0.0057	8.27E-07	240.70
40	ewe372-C4	0.03015	0.03387	0.0323	0.0381	0.03261	3.59E-06	0.0297	4.31E-06	46.51
41	ewe372-C5	0.03221	0.03299	0.0344	0.0398	0.01906	2.1E-06	0.0133	1.93E-06	103.98
42	ewe372-C8	0.03915	0.04011	n.d.	n.d.	0.140	1.54E-05	n.d.	n.d.	n.d.
43	ewe372-D11	0.02329	0.03616	0.0243	0.0311	2.231	0.000245	1.6808	0.000244	0.82
44	ewe372-D12	0.04246	0.04771	n.d.	n.d.	0.723	7.95E-05	n.d.	n.d.	n.d.
45	ewe372-D3-S	0.05643	0.2061	n.d.	n.d.	128.760	0.01416	n.d.	n.d.	n.d.
46	ewe372-D3-W	0.04717	0.1936	n.d.	n.d.	63.490	0.00698	n.d.	n.d.	n.d.
47	ewe372-D7	0.03223	0.0365	0.0286	0.0329	0.01039	1.14E-06	0.0062	8.99E-07	220.7
48	ewe372-D9	0.08502	0.2127	n.d.	n.d.	1.470	0.00016	n.d.	n.d.	n.d.
49	ewe372-F3	0.03983	0.05432	n.d.	n.d.	0.00339	3.73E-07	n.d.	n.d.	n.d.
50	ewe372-F6	0.01995	0.0313	0.0264	0.0284	2.011	0.000221	0.6098	8.84E-05	2.24
51	ewe372-G2	0.02719	0.03091	n.d.	n.d.	2.236	0.000245	n.d.	n.d.	n.d.
52	ewe372-H1-S	0.0382	0.1002	0.0282	0.0467	0.00515	5.67E-07	0.0057	8.26E-07	239.7
53	ewe372-H1-W	0.0364	0.09968	0.0283	0.0416	0.00508	5.59E-07	0.0030	4.35E-07	454.4
54	ewe374-C9	0.1311	2.049	n.d.	n.d.	4.238	0.000466	n.d.	n.d.	n.d.
55	ewe374-E6	0.3193	2.255	n.d.	n.d.	7.6778	0.000844	n.d.	n.d.	n.d.
56	ewe374-F7	0.01272	0.01367	n.d.	n.d.	7.641	0.000840	n.d.	n.d.	n.d.
57	ewe375-B8	0.1135	0.2739	n.d.	n.d.	0.946	0.000104	n.d.	n.d.	n.d.
58	ewe375-D2	0.03781	18.38	n.d.	n.d.	151.6152	0.016678	n.d.	n.d.	n.d.
59	ewe375-D4	0.04593	0.06574	0.0274	0.0362	0.00619	6.81E-07	0.0031	4.5E-07	446.3
60	ewe375-G7	11.9	3.429	n.d.	n.d.	20.020	0.002202	n.d.	n.d.	n.d.
61	ewe375-H4	0.03662	0.1174	0.0220	0.0343	0.114	1.25E-05	0.0669	9.7E-06	20.4

**Table 3:** Overview of the V-gene distribution for all 61 neutralizing antibodies selected for further analysis. The V-gene analysis was performed using VBASE2 (www.vbase2.org)^80^. This data was generated in a PhD thesis^84^.

#	Name antibody clone	VH			VL	
		V	D	J	V	J
1	ewe191-A7	IGHV3-33*01	IGHD6-13*01	IGHJ4*02	IGLV1-47*01	IGLJ3*01
2	ewe191-A9	IGHV3-21*01	IGHD6-6*01inv	IGHJ3*02	IGLV1-44*01	IGLJ3*01
3	ewe191-C1	IGHV1-3*01	IGHD3-10*01	IGHJ6*02	IGKV3-15*01	IGKJ4*01
4	ewe191-C10	IGHV3-21*01	IGHD2-21*02	IGHJ4*02	IGLV3-21*02	IGLJ3*01
5	ewe191-C11	IGHV3-33*01	IGHD6-13*01	IGHJ4*02	IGLV1-47*01	IGLJ3*01
6	ewe191-C12	IGHV5-51*01	IGHD3-10*02	IGHJ5*02	IGLV1-44*01	IGLJ3*01
7	ewe191-D1	IGHV3-21*01	IGHD2-21*02	IGHJ4*02	IGLV1-47*02	IGLJ3*01
8	ewe191-E12	IGHV5-51*01	IGHD3-16*01	IGHJ5*02	IGLV1-44*01	IGLJ1*01
9	ewe191-H10	IGHV3-21*01	IGHD2-21*02	IGHJ4*02	IGLV3-21*02	IGLJ3*02
10	ewe191-H8	IGHV3-23*04	IGHD3-10*01	IGHJ4*02	IGLV1-44*01	IGLJ3*02
11	ewe192-A6	IGHV4-39*01	IGHD6-6*01	IGHJ6*02	IGKV3-15*01	IGKJ5*01
12	ewe192-B5(P)	IGHV4-34*01	IGHD4-17*01	IGHJ2*01	IGKV1D-39*01	IGKJ4*01
13	ewe192-C11	IGHV3-21*01	IGHD5-24*01	IGHJ3*02	IGLV8-61*01	IGLJ3*01
14	ewe192-D7	IGHV3-21*01	IGHD5-24*01	IGHJ3*02	IGLV2-23*03	IGLJ3*02
15	ewe192-D8	IGHV4-39*01	IGHD6-6*01	IGHJ6*02	IGLV3-21*02	IGLJ3*02
16	ewe192-E4	IGHV4-39*01	IGHD6-6*01	IGHJ6*02	IGKV3-20*01	IGKJ4*01
17	ewe192-F11	IGHV4-39*01	IGHD6-6*01	IGHJ6*02	IGLV1-40*01	IGLJ3*01
18	ewe192-F6	IGHV4-39*01	IGHD6-6*01	IGHJ6*02	IGKV3-20*01	IGKJ1*01
19	ewe192-G12	IGHV4-39*01	IGHD6-6*01	IGHJ6*02	IGKV3-15*01	IGKJ3*01
20	ewe192-H11	IGHV3-21*01	IGHD6-6*01inv	IGHJ3*02	IGLV2-11*01	IGLJ3*02
21	ewe192-H7	IGHV3-21*01	IGHD6-6*01inv	IGHJ3*02	IGLV2-23*03	IGLJ3*02
22	ewe192-H8	IGHV4-39*01	IGHD6-6*01	IGHJ6*02	IGKV3D-15*01	IGKJ5*01
23	ewe371-A2	IGHV3-33*01	IGHD2-2*01inv	IGHJ3*02	IGLV2-14*04	IGLJ3*01
24	ewe371-A6	IGHV5-51*01	IGHD3-10*02	IGHJ5*02	IGLV3-21*01	IGLJ3*01
25	ewe371-B12	IGHV3-33*01	IGHD6-13*01	IGHJ4*03	IGLV1-47*01	IGLJ3*01
26	ewe371-D10	IGHV5-51*01	IGHD3-10*02	IGHJ5*02	IGLV1-44*01	IGLJ3*02
27	ewe371-D4	IGHV5-51*01	IGHD3-10*02	IGHJ5*02	IGLV3-19*01	IGLJ3*01
28	ewe371-D7	IGHV5-51*01	IGHD3-10*02	IGHJ5*02	IGLV1-44*01	IGLJ3*01
29	ewe371-E4	IGHV3-23*01	IGHD3-10*01	IGHJ4*02	IGLV2-14*04	IGLJ1*01
30	ewe371-E7	IGHV5-51*01	IGHD2-2*01	IGHJ5*02	IGLV2-8*01	IGLJ3*01
31	ewe371-G2	IGHV5-51*01	IGHD3-10*02	IGHJ5*02	IGLV1-44*01	IGLJ1*01
32	ewe371-G4	IGHV4-34*01	IGHD5-12*01	IGHJ6*02	IGLV1-44*01	IGLJ3*02
33	ewe371-G8	IGHV1-3*01	IGHD2-2*01	IGHJ6*02	IGLV1-44*01	IGLJ3*02
34	ewe371-H1	IGHV3-23*04	IGHD3-10*01	IGHJ4*02	IGLV1-44*01	IGLJ3*01
35	ewe371-H9	IGHV5-51*01	IGHD3-10*02	IGHJ5*02	IGLV1-44*01	IGLJ3*02
36	ewe372-A12	IGHV1-3*01	IGHD6-19*01	IGHJ3*01	IGKV1-27*01	IGKJ4*01
37	ewe372-A8	IGHV1-69*04	IGHD6-13*01	IGHJ3*02	IGKV1D-39*01	IGKJ3*01
38	ewe372-C10-S	IGHV4-34*01	IGHD2-2*02	IGHJ2*01	IGKV1D-39*01	IGKJ4*01
39	ewe372-C10-W	IGHV4-34*01	IGHD2-2*02	IGHJ2*01	IGKV1D-39*01	IGKJ4*01
40	ewe372-C4	IGHV1-69*04	IGHD1-1*01	IGHJ4*02	IGKV1D-39*01	IGKJ4*01
41	ewe372-C5	IGHV1-69*04	IGHD2-2*02	IGHJ4*02	IGKV1D-39*01	IGKJ4*01
42	ewe372-C8	IGHV1-69*04	IGHD2-2*02	IGHJ4*02	IGKV1D-39*01	IGKJ4*01
43	ewe372-D11	IGHV1-18*01	IGHD3-10*01	IGHJ6*02	IGKV3-20*01	IGKJ4*01
44	ewe372-D12	IGHV1-69*04	IGHD2-2*02inv	IGHJ4*02	IGKV1D-39*01	IGKJ4*01
45	ewe372-D3-S	IGHV4-34*02	IGHD2-15*01	IGHJ4*02	IGKV3-15*01	IGKJ1*01
46	ewe372-D3-W	IGHV4-34*02	IGHD2-15*01	IGHJ4*02	IGKV3-15*01	IGKJ1*01
47	ewe372-D7	IGHV1-69*04	IGHD2-21*02	IGHJ4*02	IGKV1D-39*01	IGKJ4*01
48	ewe372-D9	IGHV1-46*03	IGHD1-20*01	IGHJ4*02	IGKV1D-33*01	IGKJ5*01
49	ewe372-F3	IGHV1-46*03	IGHD1-20*01	IGHJ4*02	IGKV1-12*02	IGKJ4*01
50	ewe372-F6	IGHV3-23*04	IGHD4-17*01inv	IGHJ6*02	IGKV1-5*03	IGKJ5*01
51	ewe372-G2	IGHV1-69*06	IGHD2-15*01	IGHJ4*02	IGKV1D-39*01	IGKJ4*01
52	ewe372-H1-S	IGHV4-34*02	IGHD4-17*01	IGHJ2*01	IGKV1D-39*01	IGKJ4*01
53	ewe372-H1-W	IGHV4-34*02	IGHD4-17*01	IGHJ2*01	IGKV1D-39*01	IGKJ4*01
54	ewe374-C9	IGHV4-34*01	IGHD5-12*01	IGHJ6*02	IGLV1-44*01	IGLJ3*01
55	ewe374-E6	IGHV4-34*01	IGHD5-18*01	IGHJ6*02	IGLV3-19*01	IGLJ1*01
56	ewe374-F7	IGHV3-21*01	IGHD6-6*01inv	IGHJ3*02	IGLV2-23*03	IGLJ3*02
57	ewe375-B8	IGHV5-51*01	IGHD1-7*01	IGHJ5*02	IGKV3-20*01	IGKJ3*01
58	ewe375-D2	IGHV4-39*01	IGHD6-6*01	IGHJ6*02	IGKV3-20*01	IGKJ5*01
59	ewe375-D4	IGHV4-34*02	IGHD4-17*01	IGHJ2*01	IGKV1D-39*01	IGKJ4*01
60	ewe375-G7	IGHV1-69*04	IGHD1-26*01	IGHJ4*02	IGKV1D-39*01	IGKJ3*01
61	ewe375-H4	IGHV5-51*01	IGHD1-7*01	IGHJ5*02	IGKV3-20*01	IGKJ3*01

### DT binding in scFv-Fc format determined by titration ELISA

**Figure 4:**
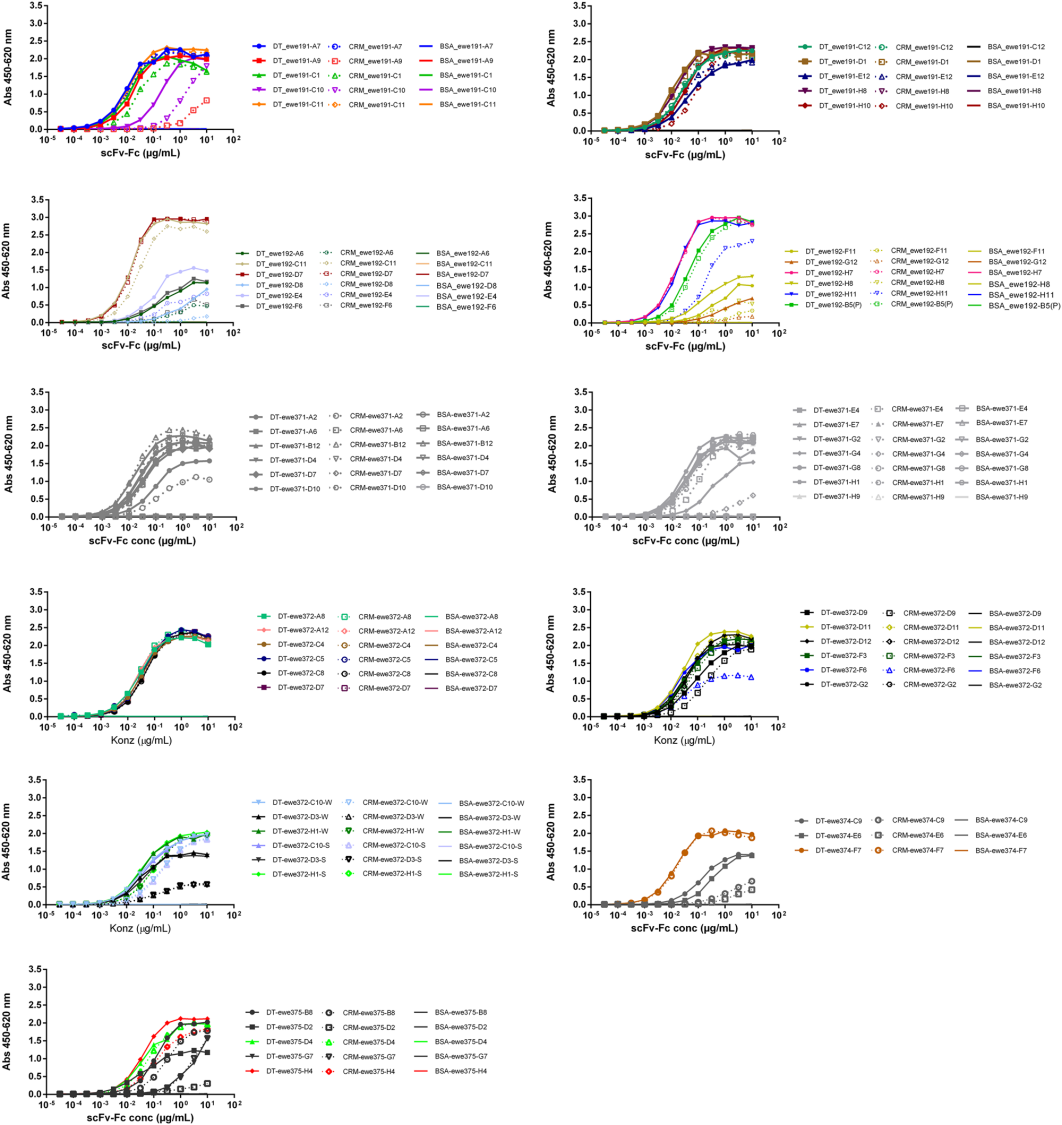
Titration ELISA for all 61 scFv-Fc antibodies binding to diphtheria toxin (filled symbol, solid line) and CRM197 (non-toxic mutant; open symbol, dotted line) with BSA as negative control (line). Detected with goat α human Fc antibody HRP conjugate, visualized with TMB substrate and measured at 450 nm with 620 nm reference. Figure modified from PhD thesis^84^.

The half maximal effective concentration (EC50) was determined for the selected 61 neutralizing scFv-Fc antibodies by performing a titration ELISA on immobilized DT and the non-toxic mutant of DT, CRM197 which contains a point mutation in the catalytic domain^48,49^ (Fig. 4 and Table 2). The EC50 for DT ranged widely from 0.0086 µg/mL to 11.9 µg/mL. The non-toxic mutant CRM197 showed binding in the range of 0.0108 µg/mL to 18.4 µg/mL. Out of 61 scFv-Fc antibodies, 16 showed more than 3-fold decreased binding to the non-toxic DT-mutant CRM197 compared to DT.

### DT binding in IgG format determined by titration ELISA

**Figure 5:**
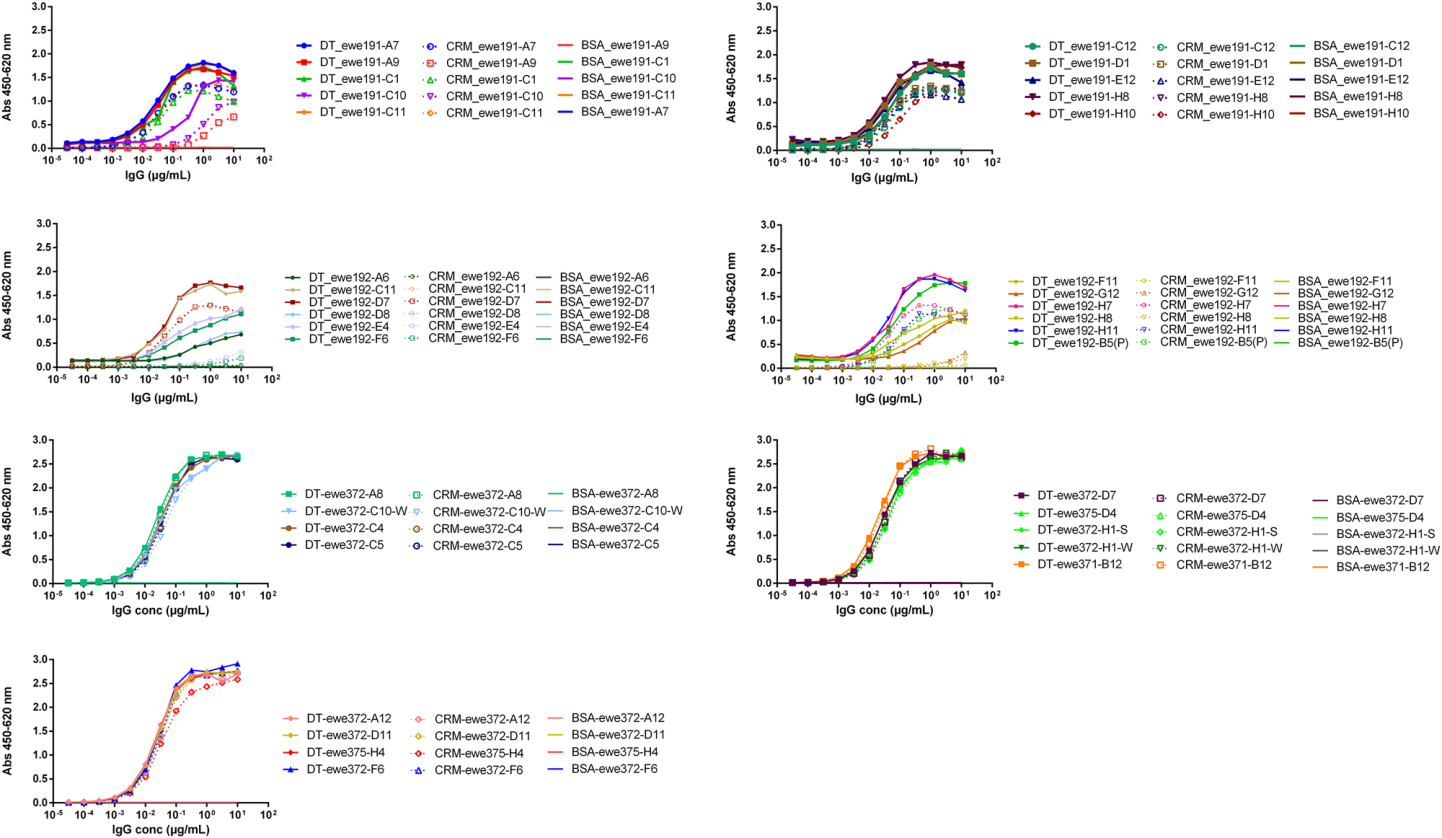
Titration ELISA for 35 IgG antibodies binding to diphtheria toxin (filled symbol, solid line) and CRM197 (non-toxic mutant; open symbol, dotted line) with BSA as negative control (line). Detected with goat α human Fc antibody HRP conjugate, visualized with TMB substrate and measured at 450 nm with 620 nm reference. Figure modified from PhD thesis^84^.

38 neutralizing scFv-Fc antibodies were cloned into the pCSEH1c (heavy chain) and pCSL3l/pCSL3k (light chain lambda/kappa) vectors^37^ for production as human IgG1. The antibodies were chosen because of their neutralization efficacy as scFv-Fc but from antibodies which very similar CDRs only one representing antibody was chosen. Three of the antibodies were not producible in IgG format (ewe372-C10-S, ewe372-F3 and ewe374-F7). The remaining 35 IgG were titrated by ELISA against DT and CRM197 (Fig. 5). All IgG antibodies bound DT, but some antibodies showed a reduced affinity binding the non-toxic mutant CRM197. The EC50 ranged from 0.0182 µg/mL to 0.523 µg/mL for DT and from 0.0214 µg/mL to 40.08 µg/mL for CRM197 (Table 2). Out of 35 IgG antibodies, 8 showed more than 3-fold decreased binding to CRM197 compared to DT. DT binding by IgG differed from the corresponding scFv-Fc significantly only for a few antibodies. One IgG gained more than 3-fold EC50 (ewe192-D8) compared to the scFv-Fc format and two antibodies lost 3-fold EC50.

### *In vitro* neutralization in IgG format

**Figure 6:**
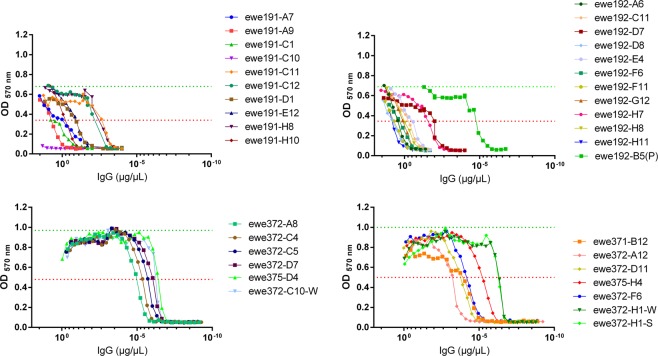
Cell based neutralization assay for 35 IgG antibodies using a toxin dose level of 4 × MCD. Untreated Vero cells are given as green dotted line. The threshold for neutralization is given as red dotted line (50% of untreated Vero cells). Figure modified from PhD thesis^84^.

The 35 IgG antibodies were tested for their ability to neutralize DT in the Vero cell TNT assay^47^ (Fig. 6). The MED50% of all purified IgG antibodies was between 3.0 pM and 1.4 µM at a constant amount of DT (4x MCD) (Table 2). Only one antibody (ewe191-C10) lost complete neutralization capacity after conversion to the IgG format. Interestingly, 11 IgG had at least a 3-fold enhancement of the MED50% compared to the corresponding scFv-Fc. The antibody ewe191-A7 showed a more than 100x improved MED50% but the initial *in vitro* neutralization efficacy of this antibody was low. The MED50% did not change more than 3-fold for the antibodies with a high neutralization efficacy except for ewe192-B5(P) which showed a 10-fold improvement.

**Figure 7:**
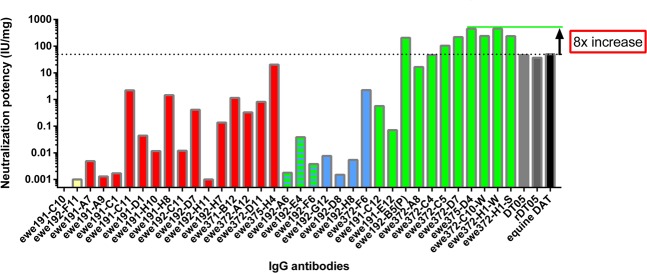
Neutralization potency of IgG antibodies expressed as IU/mg determined by Vero cell neutralization assay with a toxin dose level of 4 × MCD. All IgGs analyzed are colour coded corresponding to their DT binding domain (red = C-domain, green = R-domain, blue = T-domain, green/blue-striped = B-fragment, yellow = unknown domain). The rat hybridoma antibody DT05 and corresponding recombinant IgG rDT05 are represented in grey (and also targets the R-domain). The equine diphtheria anti toxin serum (DAT) is represented in black.

Neutralizing potency was expressed in International Units (IU) per mg by comparison with the WHO International Standard for Diphtheria Antitoxin, Equine (NIBSC code 17/230). The results are summarized in Fig. 7. The monoclonal antibodies showed a neutralization potency up to 455 IU/mg (Table 2). By comparison, a rat hybridoma antibody (DT05) and the corresponding recombinant version (rDT05) had a neutralization potency of 46.4 IU/mg and 36.6, IU/mg respectively. The neutralizing potency of a batch of equine DAT was also determined and, although not directly comparable, the specific activity was estimated to be approximately 50 IU/mg (based on total protein concentration of the purified antiserum). As an additional experiment, the best neutralizing antibodies were combined, but no synergistic effect was observed in the Vero cell assay using a DT dose of 4 × MCD (data not shown).

### Domain mapping

To identify the individual domain targeted by the 35 antibodies, the A- and B-fragments, as well as the receptor binding (R) and translocation (T) domains alone, were produced in *E. coli* as recombinant proteins. Binding to the A- and B-fragments was first tested in scFv-Fc format by immunoblot (data not shown). For antibodies that were shown to bind the B-fragment, the antibody was further tested also on the R- and T-domains. The results are given in Table 4.

**Table 4:** Analysis of the bound domains and minimal epitope region (MER) for 35 neutralizing DT antibodies. The domain mapping was performed by SDS-PAGE, followed by western blot and immunostaining with the neutralizing antibody. The MERs were defined by selection of DT single gene phage display libraries on the neutralizing antibodies followed by DNA sequencing of the selected DT fragment phage particles. The domain mapping data was generated in a PhD thesis^84^.

#	Name antibody clone	Domain mapping by immunoblot	Phage display		
			DT region (aa position)	MER (minimal epitope region)	Domain according to MER
1	ewe191-A7	C-domain	98–128	NAETIKKELGLSLTEPLMEQVGTEEFIKRFG	C-domain
2	ewe191-A9	C-domain	—	inconclusive	—
3	ewe191-C1	C-domain	—	inconclusive	—
4	ewe191-C10	n.d.	—	not analyzed	—
5	ewe191-C11	C-domain	96–124	NAETIKKELGLSLTEPLMEQVGTEEFIKR	C-domain
6	ewe191-C12	R-domain	—	inconclusive	—
7	ewe191-D1	C-domain	98–130	NAETIKKELGLSLTEPLMEQVGTEEFIKRFGDG	C-domain
9	ewe191-H10	C-domain	98–130	NAETIKKELGLSLTEPLMEQVGTEEFIKRFGDG	C-domain
10	ewe191-H8	C-domain	—	inconclusive	—
11	ewe192-A6	B-fragment	—	inconclusive	—
12	ewe192-B5(P)	R-domain	—	inconclusive	—
13	ewe192-C11	C-domain	—	inconclusive	—
14	ewe192-D7	C-domain	99–130	AETIKKELGLSLTEPLMEQVGTEEFIKRFGDG	C-domain
15	ewe192-D8	n.d.	210–223	DVIRDKTKTKIESL	T-domain
16	ewe192-E4	B-fragment	—	inconclusive	—
17	ewe192-F11	n.d.	—	inconclusive	—
18	ewe192-F6	B-fragment	—	inconclusive	—
19	ewe192-G12	n.d.	194–230	SVGSSLSCINLDWDVIRDKTKTKIESLK EHGPIKNKM	T-domain
20	ewe192-H11	C-domain	—	inconclusive	—
21	ewe192-H7	C-domain	99–127	AETIKKELGLSLTEPLMEQVGTEEFIKRF	C-domain
22	ewe192-H8	B-fragment	177–210	SVGSSLSCINLDWDVIRDKTKTKIESLKEHGPIK	T-domain
25	ewe371-B12	C-domain	99–123	AETIKKELGLSLTEPLMEQVGTEEF	C-domain
36	ewe372-A12	C-domain	—	no binding phage particles in screening ELISA	—
37	ewe372-A8	R-domain	436–471	TIPGKLDVNKSKTHISVNGRKIRMRCRAIDG DVTFC	R-domain
39	ewe372-C10-W	R-domain	—	no binding phage particles in screening ELISA	—
40	ewe372-C4	R-domain	457–473	IRMRCRAIDGDVTFCRP	R-domain
41	ewe372-C5	R-domain	436–473	TIPGKLDVNKSKTHISVNGRKIRMRCRAIDG DVTFCRP	R-domain
43	ewe 372-D11	C-domain	1–146	GADDVVDSSKSFVMENFSSYHGTKPGYVD SIQKGIQKPKSGTQGNYDDDWKGFYSTDN KYDAAGYSVDNENPLSGKAGGVVKVTYPG LTKVLALKVDNAETIKKELGLSLTEPLMEQV GTEEFIKRFGDGASRVVLSLPFAEGSSS	C-domain
47	ewe372-D7	R-domain	437–472	IPGKLDVNKSKTHISVNGRKIRMRCRAIDGD VTFCR	R-domain
49	ewe372-F3	R-domain	—	inconclusive	—
50	ewe372-F6	T-domain	—	inconclusive	—
52	ewe372-H1-S	R-domain	378–403	PAYSPGHKTQPFLHDGYAVSWNTVED	R-domain
53	ewe372-H1-W	R-domain	383–401	GHKTQPFLHDGYAVSWNTV	R-domain
59	ewe375-D4	R-domain	371–394	PAYSPGHKTQPFLHDGYAVSWNTV	R-domain
61	ewe375-H4	C-domain	1–162	GADDVVDSSKSFVMENFSSYHGTKPGYVDS IQKGIQKPKSGTQGNYDDDWKGFYSTDNKY DAAGYSVDNENPLSGKAGGVVKVTYPGLTK VLALKVDNAETIKKELGLSLTEPLMEQVGTEE FIKRFGDGASRVVLSLPFAEGSSSVEYINNW EQAKALSVE	C-domain

According to the domain mapping by immunoblot, 10 antibodies bound the R domain, 15 antibodies bound the C-domain and 1 antibody bound the T-domain. 4 antibodies bound the B-fragment, but did not bind separately to either the T- or R-domain alone, and for 4 antibodies it was not possible to determine the domain. These results showed that the majority of neutralizing antibodies were targeting the R domain. The best neutralizing antibodies against the R-domain were ewe375-D4 and ewe372-C10-W, the best IgG against the C-domain was ewe375-H4, and the best neutralizing antibody binding the T-domain was ewe372-F6. The antibody ewe372-C10-W was excluded from further analysis because the size exclusion chromatography (SEC) profile showed an increased formation of dimers (data not shown).

### Epitope mapping using single gene phage display

**Figure 8:**
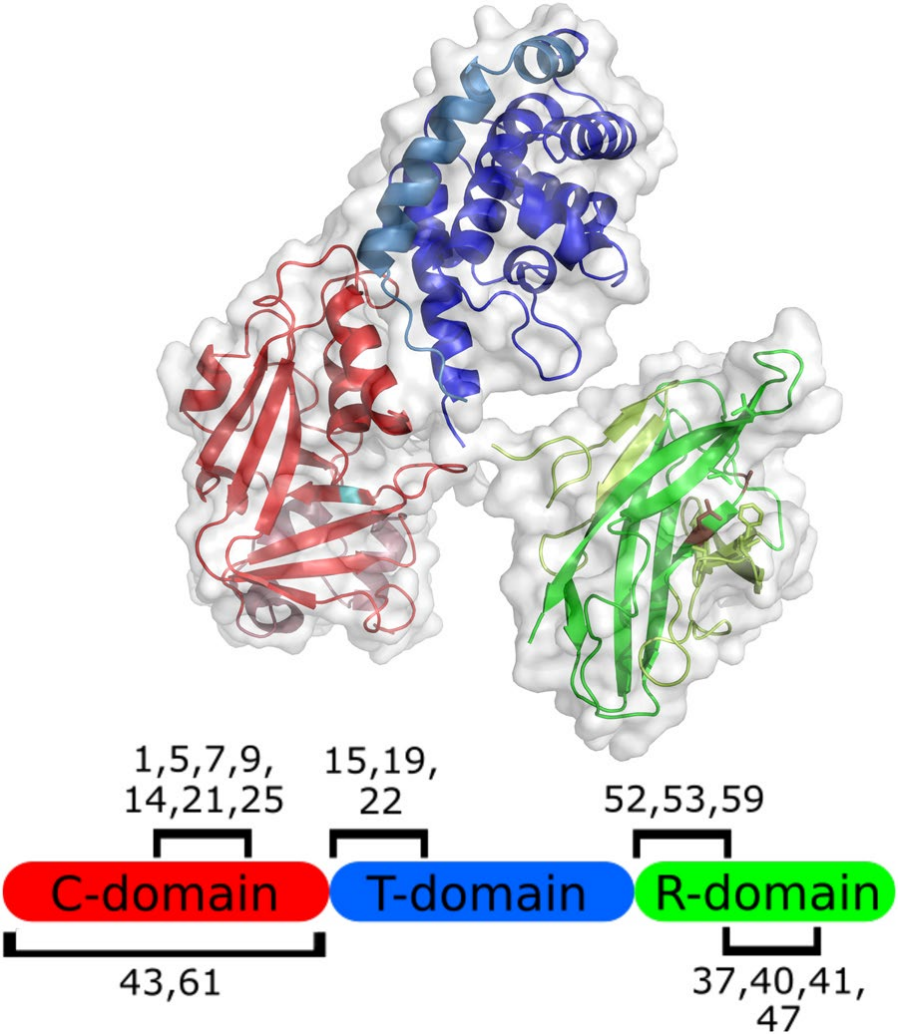
Overview of the identified minimal epitope regions (MERs) for 19 antibodies analyzed using single gene phage display. A) schematic overview and B) 3D based on pdb 1ddt, the MERs are highlighted. The antibody number is represented as shown in Table 4.

To identify the epitopes, the DNA for the B-fragment and the sequence encoding the C- and T-domains were fragmented and two independent phage display libraries were constructed by cloning into the ORFeome phage display vector pHORF3^50^ followed by packaging with Hyperphage for ORF selection to improve library quality. The library quality and the coverage of the complete DT gene was analyzed by sequencing of approximately 80 clones (Supplementary Fig. 1) showing a nearly complete coverage of DT using this limited amount of sequences. We expected to get also overlapping C-T fragments and were surprised to get the most fragments at the beginning of the C or T domain. Subsequently, a panning was performed on 34 of the 35 best neutralizing antibodies in the scFv-Fc format using the A- and the B-fragment libraries separately. We excluded ewe191-C10 because this antibody lost neutralization capacity after conversion to the IgG format. Individual epitope phage clones from the second panning round were screened by ELISA. For 19 antibodies, the minimal epitope region (MER) was identified (Table 4). An overview of all identified MERs is given in Fig. 8 and the detailed sequencing results for each identified MER are given in Supplementary Fig. 2A–S. For two antibodies no binding partners were identified in the screening ELISA. For 13 antibodies the data were inconclusive because DT fragments of two epitope regions were selected and identified in the screening ELISA (e.g. for ewe192-F11 in Supplementary Fig. 2T), or clones from one epitope region were identified but, in addition, more than one outlier in other DT regions or inverse clones were selected. For ewe372-H1-S, the outlier may be excluded because of the results derived from ewe372-H1-W which differs only by one amino acid in framework one. The length of the identified MERs ranged from 14 amino acids for ewe192-D8 to the complete C-domain for ewe372-D11 ewe374-H4. The domains identified using the immunoblot approach were always in accordance with the domains identified by epitope mapping.

The combined results of the immunoblot and the phage display approach suggest that 15 antibodies bound the C-domain, 11 bound the R-domain, 4 bound the T-domain, and for one antibody no domain was assigned. For 3 antibodies, only the complete B fragment was identified using both approaches.

### Affinity determination

Six anti-DT antibodies in the scFv-Fc and IgG format were further characterized by Microscale Thermophoresis (MST) measurements to determine their EC50 value in solution. Here, we focused on the antibodies ewe191-A7 and ewe191-C12 as well as ewe192-B5(P) as the best neutralizing antibodies derived from MTP panning. Furthermore, all three antibodies derived from panning in solution (ewe372-F6, ewe375-D4 and ewe375-H4) that were later tested in guinea pig *in vivo* protection assays were included in this analysis. The results are given in Table 5 and Supplementary Figure 3. The EC50 values of the antibodies in IgG format were quite similar, ranging from 9.4 nM to 13.6 nM. In the scFv-Fc format, the measured EC50 values were more diverse. For half of the antibodies the EC50 value decreased to 3–7 nM, and for the other half it increased to 20–40 nM. The observed difference in the formats might be due to the altered flexibility of the antibody arms having an influence on the avidity of the antibody. This avidity behavior was also visible in the MST measurement in the form of a secondary binding event for ewe191-A7, ewe191-C12, ewe372-F6 and ewe375-H4 in both formats.

**Table 5:** Affinity measurement for selected anti-DT antibodies in scFv-Fc and IgG format. All measurements were performed in triplicate in PBS. *Here the secondary binding event was not as prominent.

Antibody clone	scFv-Fc EC50 [nM]	IgG EC50 [nM}	Secondary binding event
ewe191-A7	19.7 +/− 2.6	9.4 +/− 2.2	yes
ewe191-C12	3.1 +/− 0.6	10.7 +/− 2.2	yes
ewe192-B5(P)	40.5 +/− 1.8	12.8 +/− 1.1	no
ewe372-F6	6.8 +/− 2.6	11.5 +/− 1.1	yes
ewe375-D4	22.8 +/− 7.1	12.6 +/− 1.2	no
ewe375-H4	6.4 +/− 0.9	13.6 +/− 1.5	yes*

### *In vitro* neutralization using higher toxin concentrations and the effect of using antibody combinations

The antibodies ewe375-D4, ewe375-H4 and ewe372-F6 selected for *in vivo* protection experiments were further analyzed in the Vero cell TNT assays^47^ using higher toxin concentrations. These *in vitro* assays were performed at a range of (increasing) DT dose levels referred to as Lcd/1000, Lcd/100 and Lcd/10. The amount of toxin needed to cause more than 50% cell death in the presence of a fixed, defined amount of reference DAT is given as the limit of cytopathic dose (Lcd). The Lcd number refers to the inverse of the antitoxin concentration in IU. Hence, Lcd/1 is the amount of toxin per mL causing >50% cell death in the presence of 1 IU DAT, Lcd/10 for 0.1 IU DAT, and so forth. In our assay, Lcd/1000 equates to approximately 40 × MCD. In addition, neutralization using 4 × MCD was measured in parallel again for direct comparison. The sensitivity of cells to DT is given as multiple of the minimum cytopathic dose (MCD) – so the assay performed at 4 × MCD uses a fixed DT concentration that is 4 × higher than the minimum concentration causing >50% cell death. The results for individual antibodies and antibody combinations are given in Table 6. The results for the neutralization assay using 4 × MCD are, as expected, in the same range as the results given in Table 2. There was no loss of potency when using Lcd/1000 (~40 × MCD), but the individual antibodies lost neutralization capacity at higher DT dose levels of Lcd/100 or Lcd/10. However, strong neutralizing activity was observed when antibodies were used in a combination of two or three, giving a specific activity ranging from 80–160 IU/mg (Table 6).

**Table 6:** *In vitro* neutralization at different toxin dose levels for individual antibodies and antibody combinations in the Vero cell TNT assay. The neutralization potency is reported as IU/mg. The assay using 4xMCD DT is an independent experiment from the assay in Table 2. n.d. = not done.

Name antibody clone	4 × MCD [IU/mg]	Lcd/1000 [IU/mg]	Lcd/100 [IU/mg]	Lcd/10 [IU/mg]
ewe375-D4 (anti R-Domain)	445.7	446	<20	<2.8
ewe375-H4 (anti C-Domain)	40.5	41	<20	<3.2
ewe372-F6 (anti T-domain)	4.4	n.d.	<20	<3.1
ewe375-D4 + ewe375-H4	n.d.	n.d.	160	100
ewe375-D4 + ewe372-F6	n.d.	n.d.	160	100
ewe375-H4 + ewe372-F6	n.d.	n.d.	80	100
ewe375-D4 + ewe375-H4 + ewe372-F6	n.d.	n.d	80	100

### Analysis of *in vivo* neutralization by intradermal challenge and the effect of antibody combinations

The *in vivo* neutralization efficacy of the antibodies was analyzed in a non-lethal intradermal challenge assay in guinea pigs using a DT dose of Lr/100^51,52^. Lr/100 is defined as the smallest amount of DT that causes a weak but detectable erythema in the presence of 0.01 IU of reference DAT antitoxin. The Lcd/100 level of DT in the *in vitro* TNT Vero cell assay is analogous to Lr/100 in the *in vivo* intradermal challenge assay, showing that the *in vitro* assay may be a suitable replacement for the *in vivo* test. The results are given in Table 7. The individual antibodies did not show a high level of neutralization, whereas all antibody pairs and the triple antibody combination resulted in a neutralization potency of 79 IU/mg.

**Table 7:** *In vivo* neutralization (at Lr/100 toxin dose level) for individual antibodies and antibody combinations in the guinea pig intradermal challenge assay. The neutralization potency is reported as IU/mg.

Name antibody clone	[IU/mg]
ewe375-D4 (anti R-domain)	<0.72
ewe375-H4 (anti C-domain)	<6.4
ewe372-F6 (anti T-domain)	<1.23
ewe375-D4 + ewe375-H4	79.4
ewe375-D4 + ewe372-F6	79.4
ewe375-H4 + ewe372-F6	79.4
ewe375-D4 + ewe375-H4 + ewe372-F6	79.4

## Discussion

We describe here the development of recombinant human monoclonal antibodies that neutralize DT and have the potential to be developed further as a therapeutic alternative to equine DAT. The use of recombinant antibodies is the prevailing approach for the development of therapeutic antibodies^53–55^. The advantages include a sequence defined human antibody produced under controlled and reproducible conditions in mammalian cell culture as opposed to an animal derived blood product with batch-to-batch variation and the risk of adverse reaction in the form of serum sickness.

Human recombinant antibodies were selected from two different immune libraries derived from immunized donors. The first library was constructed from all PBMCs while the second was derived only from CD138^+^ plasma cells. Two panning approaches were performed using both libraries. Panning on immobilized antigen and panning in solution were both performed because the output using both strategies may differ due to different ways an antigen is presented, and to mitigate the risk of denaturation by MTP immobilization^56^. In order to improve the throughput, for the clones enriched by panning in solution, a functional screening on DT neutralization was performed to directly identify neutralizing antibodies, instead of identification of binding followed by neutralization. The observed V-gene distribution of the DT neutralizing antibodies matched the distribution of V-genes from naive antibody gene libraries^57^ with the exception VH4-34. This V-gene is rarely selected from phage display libraries (based on our in-house data from >3500 phage display selected and validated scFv) but is common in the natural human antibody repertoire^58^. This V-gene is also described as self-reactive to both carbohydrate structures of human erythrocytes and commensal bacteria^59^. Interestingly, both of the best neutralizing antibodies directed to the R- domain (see below) have the VH4-34 V-gene.

The binding of the neutralizing antibodies to DT and the mutant CRM197^48,49^ was analyzed and reduced binding to CRM197 was seen for many of the selected antibodies. This result is in accordance with Bigio *et al*.^60^ who found, that the conformation of CRM197, which has only one amino acid change in the catalytic center, is not identical to wildtype DT. The antibodies were initially analyzed in the scFv-Fc format because the conversion from scFv to scFv-Fc is only one cloning step^61^. For further *in vitro* and *in vivo* assays, 35 selected antibodies were converted to the IgG format because IgGs are the preferred format for therapeutic antibodies^54^. Here, differences in the EC50 between the scFv-Fc format and IgG were measured. A few antibodies lost affinity by cloning from scFv to IgG format, possibly due to different folding or the reduced flexibility of the angle between VH and VL in the Fab/IgG compared to the scFv format because of the stabilization by CH1+ CL^37,62,63^. Conversely, the affinity was improved for the antibody ewe192-D8 whose initially weak EC50 increased by around 10-fold following conversion to IgG. The EC50 values determined by MST differed to that measured in ELISA by a factor of ~100. In ELISA the analyzed antibodies had an EC50 value of under 1 nM. The interaction in ELISA is measured on immobilized DT and not in solution which likely explains the vast difference to the MST results.

The recognized domains and minimal epitope regions of the neutralizing antibodies were determined by immunoblot or phage display. Epitope mapping by phage display^64–66^ is a good supplement to peptide arrays like PepSpots^67,68^ or microarrays^69^. While peptide arrays identify linear epitopes very precisely, conformational or non-continuous MERs can be identified by phage display. Using both approaches, the binding domain was identified for 33 antibodies, but it was not possible to identify the epitope domain for one of the antibodies. It was possible to narrow down the minimal epitope region (MER) to 14 amino acids in the best case. For two of the analyzed antibodies, the complete C-domain was selected by phage display, indicating that the epitope is not linear. Interestingly, these two antibodies bound the C-domain in western blot after SDS-PAGE, indicating sufficient refolding of the C-domain on the PVDF membrane. In the case of ewe192-H8, domain mapping revealed that only the B-fragment was the antibody target and not a specific domain within that fragment of DT. However, using the phage display approach the T-domain of the B-fragment was identified showing that the conformation of the recognized MER may depend on the formation of T- and R-domains. It was demonstrated that all three DT domains are targets of highly neutralizing recombinant antibodies which is in accordance with previous publications showing that neutralizing human recombinant antibodies^70,71^ or murine monoclonal antibodies^72^ can target all three domains.

The neutralization efficacy of the scFv-Fcs and IgGs were first analyzed *in vitro* using the Vero cell toxin neutralization assay. Approximately a third of the IgGs showed an improved *in vitro* efficacy (MED50%) compared to the corresponding scFv-Fc. To our knowledge, this is the first direct comparison of scFv-Fc and IgG in *in vitro* neutralization assays. Other publications have only compared monovalent scFv with the corresponding bivalent IgG, e.g. for neutralization of varicella-zoster virus^73^. The anti R-domain antibodies, ewe372-H1-W and ewe375-D4 had an estimated relative potency of 454 IU/mg and 446 IU/mg, respectively, at 4 × MCD. The human recombinant antibodies developed elsewhere (315C4^70^ and DTD4^71^) were reported to have a lower potency (136 IU/mg and 52 IU/mg, respectively). The *in vitro* neutralization assay using 315C4 of Sevigny *et al*.^70^ was also performed at a relatively low toxin dose level of ~4 × MCD (based on the end point quoted for the reference antitoxin), and the DT neutralization efficacy for DTD4 by Kakita *et al*.^71^ cannot be calculated in IU/mg from the data given in the publication.

The specific activity of the best neutralizing human monoclonal antibodies from our studies in the Vero cell TNT also exceeded (approximately 10-fold) the potency of a rat monoclonal antibody that was included in our study. To our knowledge, ewe372-H1-W and ewe375-D4 are the antibodies with the highest published *in vitro* DT neutralization potency.

For the *in vivo* neutralization experiments, a non-lethal intradermal challenge experiment^51,52^ was performed based on the method described in the European Pharmacopoeia. This assay is performed at a higher toxin dose (referred to as Lr/100) than that used in the routine Vero cell TNT. Surprisingly, we found that individual antibodies did not neutralize DT even when the antibody dose was increased. In this assay, the specific activity was at least 500-fold lower compared to that measured in the Vero cell TNT at 4 × MCD. These findings were confirmed by performing the Vero cell TNT at higher toxin dose levels. An equine polyclonal DAT included as a comparator in all Vero cell TNT assays had a similar neutralizing potency at all toxin dose levels used. When the monoclonal antibodies were tested in combination – either as a pair or a trio - excellent neutralization of DT was observed *in vivo* (at Lr/100) and *in vitro* (at Lcd/100 and Lcd/10). The specific activity of the antibody combinations was determined to be approximately 80 IU/mg *in vivo* and 80–160 IU/mg *in vitro*. At the low toxin dose level of 4 × MCD in Vero cells, we did not observe any synergistic effect of combining more than one antibody. We hypothesize that the mode of action is not only based on neutralization, but also on the formation of aggregates as described by Heidelberger and Kendall^74^. This mode of action was also described for other recombinant antibodies which bind more than one epitope on the targeted toxin, e.g. for the anti-TcdA antibody Actotoxumab binding to a repeating epitope structure on the toxin^75^ or bispecific VHH antibody constructs against the plant toxin ricin^76,77^. For the antibody 315C4 an *in vivo* neutralization potency using a lethal guinea pig challenge assay of 64 IU/mg was published^70^. Further *in vivo* tests were performed for the antibody S315 which is derived from 315C4, resulting in a neutralization potency which corresponds to 58.8 IU/mg^78^. An *in vivo* neutralization potency of 73.6 IU/mg were published by Kakita *et al*.^71^ for DTD4 using a rabbit skin test as *in vivo* model. Despite differences in the models used, our results suggest that the neutralizing potency of the antibody combinations tested in our study is comparable and likely superior to the results obtained for single monoclonal antibodies developed elsewhere.

The use of antibody combinations for the development of a therapeutic product is more challenging from a regulatory aspect, but may have clear advantages as a therapeutic because it mimics a polyclonal serum by binding to different epitopes. Further, the use of an antibody combination binding more than one domain on the target toxin may be a more robust therapeutic approach and may provide protection against toxin escape mutants. The antibodies described here are promising candidates for future regulatory and clinical development as an alternative to equine DAT for diphtheria therapy.

## Material and Methods

### Generation of an immune anti-DT antibody gene library

For the generation of a human immune library against DT, blood from three donors who have been regularly boost-vaccinated was collected 7 days after they received a boost vaccination of a combined diphtheria and tetanus vaccine. This was performed in accordance with the Declaration of Helsinki. All the voluntary donors were informed about the project and gave their consent. The use of blood samples for the amplification of antibody gene fragments to develop antibody phage display libraries was approved by the ethical committee of the Technische Universität Braunschweig (Ethik-Kommission der Fakultät 2 der TU Braunschweig, approval number DM-2014-08). The donors have given their consent for study publication. The library construction was performed as described previously with minor modifications^79^. In brief, the peripheral blood mononuclear cells (PBMC) were extracted from the blood via Ficoll (GE Healthcare, Freiburg, Germany) and RNA isolated with Trizol and a Trizol RNA purification kit (Zymo Research, Freiburg, Germany). For the B-cell sorted library, B-cells were stained with mouse α-CD138 (FITC conjugated) antibody (ab27390, abcam, Cambridge, UK) and sorted via FACS and taken up in Trizol. In addition, the RNA of the sorted B cells was extracted using the Trizol RNA purification kit according to the manufacturer’s instructions. The RNA was converted into cDNA using Superscript III (Thermo Fisher Scientific, Waltham, USA) according to the manufacturer’s instructions. The cDNA was amplified with specific primers for the VH chain, primers for kappa light chain and primer for lambda light chain. These PCR products were again amplified with primers adding a restriction site for further cloning into the *E. coli* expression vector pHAL51. The vector pHAL51 is derived from the vector pHAL30^57^ with an AscI stuffer in the VL cloning site. Firstly, the light chain was cloned with the restriction sites MluI and NotI, and secondly the VH chain was cloned with the restriction sites HindIII and NcoI. The efficiency of library cloning was tested with a colony PCR and the rate of complete scFv were determined. The libraries were further packaged with Hyperphage^45,46^ followed by addition of glycerine (20% (v/v)) and storage at −80 °C.

### Antibody selection in microtiter plate

The antibody selection was performed as described previously with modifications^33^. In brief, for the panning procedure, the antigen was immobilized on a Costar Highbinding 96 well plate (Corning, New York, USA). 1 µg of DT (List Biological Laboratories, Campbell, USA) was diluted in PBS (137 mM NaCl; 1.76 mM KH_2_PO_4_ × 2 H_2_O) and coated in the wells at 4 °C overnight. Next, the wells were blocked with 350 µL 2% MBPST (2% (w/v) milk powder in PBS; 0.05% Tween20) for 1 h at RT and then washed 3 times with PBST (PBS; 0.05% Tween20). Before adding the libraries to the coated wells, the libraries (5 × 10^10^ phage particles) were preincubated with 2% MPBST on blocked wells for 1 h at RT. The libraries were transferred to the coated wells, incubated for 2 h at RT and washed 10 times. Bound phage was eluted with trypsin (10 µg/mL) at 37 °C and 5 µL of eluate was used for titration. The remaining 145 µL was transferred to a 96 deep well plate (Greiner Bio-One, Frickenhausen, Germany) and incubated with 145 µL *E. coli* TG1 (OD_600_ = 0.5) firstly for 30 min at 37 °C, then 30 min at 37 °C and 650 rpm to infect the phage particles. 1 mL 2xYT-GA (1.6% (w/v) Tryptone; 1% (w/v) Yeast extract; 0.5% (w/v) NaCl (pH 7.0), 100 mM D-Glucose, 100 µg/mL ampicillin) was added and incubated for 1 h at 37 °C and 650 rpm, followed by addition of 1 × 10^10^ cfu M13KO7 helper phage. Subsequently, the infected bacteria were incubated for 30 min at 37 °C followed by 30 min at 37 °C and 650 rpm before centrifugation for 10 min at 3220 × g. The supernatant was discarded and the pellet resuspended in fresh 2xYT-AK (1.6% (w/v) Tryptone; 1% (w/v) Yeast extract; 0.5% (w/v) NaCl (pH 7.0), 100 µg/mL ampicillin, 50 µg/mL kanamycin). The phage antibodies were amplified overnight at 30 °C and 650 rpm and used for the next panning round. In total three panning rounds were performed. In each round, the stringency of the washing procedures was increased (20x in panning round 2 and 30x in panning round 3). After the third panning round, the titer plate was used to select monoclonal antibody clones for the screening ELISA.

### Production of soluble antibodies in MTPs and screening ELISA

Soluble antibody fragments (scFv) were produced in 96-well MTPs with polypropylene (U96 PP, Greiner Bio-One). Briefly, 150 μL 2xYT-GA was inoculated with the bacteria bearing scFv expressing phagemids. MTPs were incubated overnight at 37 °C and 850 rpm in a MTP shaker (Thermoshaker PST-60HL-4, Lab4You, Berlin, Germany). A volume of 140 μL 2xYT-GA in a PP-MTP well was inoculated with 10 μL of the overnight culture and grown at 37 °C and 850 rpm until bacteria reached an OD_600_ of 0.5. Bacteria were harvested by centrifugation for 10 min at 3,220 × g and the supernatant was discarded. To induce expression of the antibody genes, the pellets were resuspended in 150 μL 2xYT supplemented with 100 μg/mL ampicillin and 50 μM isopropyl-beta D thiogalacto pyranoside (IPTG) and incubated at 30 °C and 850 rpm overnight. Bacteria were pelleted by centrifugation for 10 min at 3,220 × g and 4 °C. The scFv-containing supernatant was transferred to a new PP-MTP and stored at 4 °C before ELISA analysis.

For the ELISA, 100 ng of DT was coated on 96 well microtitre plates (MaxiSorp, Thermo Fisher Scientific) in PBS (pH 7.4) overnight at 4 °C. After coating, the wells were washed three times with PBST and blocked with 2% MPBST for 1 h at RT, followed by three washing steps with PBST. Supernatants containing monoclonal scFv were mixed with 2% MPBST (1:2) and incubated in the antigen coated plates for 1.5 h at RT followed by three PBST washing cycles. Bound scFv were detected using murine mAb 9E10 which recognizes the C-terminal c-myc tag (1:50 diluted in 2% MPBST) and a goat anti-mouse serum conjugated with horseradish peroxidase (HRP) (A0168, Sigma) (1:50,000 dilution in 2% MPBST). Bound antibodies were visualized with tetramethylbenzidine (TMB) substrate (20 parts TMB solution A (30 mM Potassium citrate; 1% (w/v) Citric acid (pH 4.1)) and 1 part TMB solution B (10 mM TMB; 10% (v/v) Acetone; 90% (v/v) Ethanol; 80 mM H_2_O_2_ (30%)) were mixed). After stopping the reaction by addition of 1 N H_2_SO_4_, absorbance at 450 nm with a 620 nm reference was measured in an ELISA plate reader (Epoch, BioTek, Bad Friedrichshall, Germany). Monoclonal binders were sequenced and analyzed using VBASE2 (www.vbase2.org)^80^.

### Antibody selection in solution

For the panning in solution, the antigen was biotinylated with a biotin labelling reagent (EZ Link Sulfo-NHS-LC-Biotin, Thermo Fisher Scientific). 100 ng of the biotinylated antigen was preincubated in a 1.5 mL low protein binding tube (Eppendorf, Hamburg, Germany) with 2% BSA-PBS on an overhead shaker. After 2 h, 10 µL magnetic streptavidin beads (Dynabeads M-280, Thermo Fisher Scientific) were added to the tube and incubated for an additional 30 min. The tubes were placed for 5 min on a magnetic rack and the supernatant was discarded. The streptavidin beads were washed 10 times with 0.8 mL PBST. Bound phage was eluted with trypsin (10 µg/mL) at 37 °C and 5 µL was used for titration. The remaining 145 µL was transferred to a 96-well deep well plate and incubated with 145 µL *E. coli* TG1 (OD_600_ = 0.5) firstly for 30 min at 37 °C, then for 30 min at 37 °C and 650 rpm to infect the phage particles. 1 mL 2xYT-GA was added and incubated for 1 h at 37 °C at 650 rpm followed by addition of 1 × 10^10^ M13KO7 helper phage. Again, the infected bacteria were incubated for 30 min at 37 °C and 30 min at 37 °C and 650 rpm before a centrifugation for 10 min at 3220 × g. The supernatant was discarded and the pellet resuspended in fresh 2xYT-AK. The phage antibodies were amplified at 30 °C overnight and 650 rpm and the produced phage particles were used for the next panning round. In total three panning rounds were performed. In each round, the stringency of the washing procedures was increased (20x in panning round 2 and 30x in panning round 3). The output of the third panning rounds was used for batch cloning into the scFv-Fc format and functional screening.

### scFv-Fc and IgG antibody cloning, expression and purification

Unique scFv sequences isolated by antibody-phage display in MTPs or the complete output of the third panning rounds (all scFv encoding DNA fragments) were subcloned into pCSE2.7-hIgG1-Fc-XP using NcoI/NotI (New England Biolabs, Frankfurt, Germany) for mammalian production as scFv-Fc, an IgG-like antibody format. The vector pCSE2.7-hIgG1-Fc-XP is derived from the vector pCSE2.6-hIG1-Fc-XP^61^ with an additional AscI restriction site in the cloning site for improved NcoI/NotI cloning. The best neutralizing antibodies were converted into the human IgG1 format by subcloning of VH in the vector pCSEH1c (heavy chain) and VL in the vector pCSL3l/pCSL3k (light chain lambda/kappa)^37^. EXPI293F (Thermo Fisher Scientific) cells were transfected with both vectors in parallel. For production, the transfected EXPI293F cells were cultured in chemically defined medium F17 (Thermo Fisher Scientific) supplemented with 0.1% pluronic F68 (PAN-Biotech, Aidenbach, Germany) and 7.5 mM L-glutamine (Merck). A subsequent protein A purification was performed as described previously^61^.

### Antibody titration ELISA

The titration ELISA was performed similar to the indirect antigen ELISA (see above). A dilution series was made in blocking solution starting with 1 µg antibody. Afterwards, 100 µL of each dilution was added per well and incubated for 1 h at RT. The plate was washed 3 times with PBST. Goat α-human IgG (Fc-specific, A0170, Sigma) conjugated to horse radish peroxidase (HRP, 1:70,000) was used as the secondary detection antibody and was incubated for 1 h at RT. Unbound antibodies were removed by additional washing steps. Bound antibodies were visualized with TMB substrate (20 parts TMB solution A and 1 part TMB solution B). After stopping the reaction by adding 100 µL 1 N H_2_SO_4_, absorbance at 450 nm with a 620 nm reference was measured in an ELISA plate reader.

### PIPE cloning

Polymerase incomplete primer extension (PIPE) cloning was used to introduce a site directed mutagenesis followed by cloning into the pCSE2.7-hIgG1-XP vector. PCR was performed in 10 µL reactions using 0.5 ng of template, 0.25 µM forward and reverse primers, Q5 reaction buffer and 1 U HotStart Q5 DNA polymerase (NEB). The cycling conditions were: 98 °C 30 sec, 30 × (98 °C 10 s, 72 °C 2:30 min for products >1.5 kb or 1 min for < 1.5 kb). The correct size of the PCR products was checked with a 1% (w/v) agarose gel in TAE buffer containing 1 µg ethidium bromide. 2.5 µL vector product and 2.5 µL insert product were digested for 1 h at 37 °C with 10 U of DpnI NEB and the total amount was used to transform 20 µL of XL1 Blue MRF’ supercompetent cells (Agilent Technologies, Waldbronn, Germany) via heat shock transformation according to manufacturer’s instructions.

### Recombinant production and purification of DT fragments and domains

The gene fragments encoding the A or B fragment of DT or the single domains, were amplified from *Corynebacterium diphtheria* (DSM 43989, DSMZ) and cloned into pET21a(+) (Merck). Production of the recombinant proteins with a C-terminally 6 × His-tagged proteins in the *E. coli* BLR(DE3) expression system was performed in LB-A-IPTG (1% (w/v) tryptone; 0.5% (w/v) yeast extract; 1% (w/v) NaCl (pH 7.0), 100 µg/mL ampicillin, 0.5 mM IPTG) over 3 h at 37 °C. Recombinant proteins containing a HIS-tag were purified using immobilized metal ion affinity chromatography (IMAC). The bacterial culture was centrifuged at 10,000 × g for 15 min at 4 °C. The pellet was then resuspended in binding buffer (20 mM Tris-HCl, 0.5 M NaCl, 5 mM imidazole, and, if the protein was not soluble, 6 M urea was added) and sonicated 3 times for 2 min with pulsed cycles. Afterwards, the suspension was centrifuged at 16,000 × g for 10 min at 4 °C and the supernatant was loaded to a support column containing Sepharose (activated with 0.1 M NiSO_4_). The column was washed with washing buffer (20 mM Tris-HCl, 0.5 M NaCl, 60 mM imidazole, for some domains 6 M urea was added) until a OD_280_ of approximately 0 for the flow-through was reached. The bound antibodies were then eluted with elution buffer (20 mM Tris-HCl, 0.5 M NaCl, 1 M imidazole, for some domains 6 M urea was added). The buffer was exchanged to PBS by dialysis and the purified proteins were stored for a short time at 4 °C or aliquoted and stored at −20 °C long term.

### Cultivation of vero cells and vero cell toxin neutralization test

For *in vitro* DT Toxin neutralization assay (TNT), a Vero cell line (African green monkey kidney cells) was used. Vero cells were cultivated in EMEM medium (Biochrom) supplemented with 0.015 M HEPES (Carl Roth), 0.1% Glucose (Carl Roth), 100 µg/mL penicillin/streptomycin (Biochrom), 2 mM l-glutamine (Biochrom) and 5% fetal calf serum (Biochrom) at 37 °C and 5% (v/v) CO2, and passaged 2–3 times per week when confluency exceeded 90%.

For routine screening, the Vero cell assay was performed using a DT dose referred to as 4 × MCD. This is a fixed dose of DT at 4 × the minimum concentration causing more than 50% cytopathic effect in Vero cells. This is the most sensitive version of the assay allowing very low concentrations of antibodies to be screened for neutralization. For further analysis of IgG antibodies, assays were performed at higher toxin dose levels referred to as Lcd/1000, Lcd/100 or Lcd/10^81^. These DT dose levels are defined as the minimum concentration of DT required to produce a cytopathic effect in Vero cells after 6 days in culture when DT is mixed with a fixed concentration of a reference antitoxin at 0.1, 0.01 or 0.001 IU/ml for Lcd/10, Lcd/100 or Lcd/1000, respectively. The DT dose levels were pre-determined by titration of DT against the reference antitoxin (WHO International Standard for Diphtheria Antitoxin Equine, NIBSC code 17/230).

For the neutralization assays, serial, two-fold dilutions of antibody samples either individually (ranging from 300 µg to 0.1 pg) or in combination were made in complete EMEM in 96-well tissue culture plates, prior to addition of an equal volume (50 µL) of diphtheria toxin (TU Braunschweig: List Biological Laboratories; NIBSC: EDQM Biological Reference Preparation, batch 1) at a fixed concentration (dependent on the DT dose level of the assay). Samples were left for 1 h at room temperature to allow DT neutralization to occur followed by addition of 50 µL of a suspension of Vero cells to give a cell density of 2 × 10^4^ cells per well. Control wells were included on every plate containing DT only (toxin control) or cells only (cell control). The reference DAT antitoxin was included on every plate. Plates were incubated for 6 days at + 37 °C in humidified air supplemented with 5% CO_2_. Cell viability was then determined by incubating cells with Thiazolyl Blue Tetrazolium Bromide (MTT, Sigma). Cells were incubated with MTT (5 mg/ml in PBS) for 4 h at + 37 °C in humidified air containing 5% CO_2_. Supernatants were carefully removed and the MTT-formazan product in viable cells was extracted using 10% w/v SDS in 50% v/v dimethylformamide, pH 4.7 (100 µL/well). The plates were returned to the incubator overnight to allow for complete extraction and stabilization of the coloured product and the absorbance was read at 570 nm. End points were defined as the lowest dilution at which neutralization of DT was sufficient to produce >50% viable Vero cells (where the 50% cut-off was based on the half-maximum OD for eight control wells containing Vero cells only and eight wells using the specific, constant DT concentration).

### Domain mapping by immuno blot

Sodium dodecyl sulfate polyacrylamide gel electrophoresis (SDS-PAGE) was used to separate the DT-fragments according to their electrophoretic mobility. The DT-fragments were mixed with 5 × Laemmli’s sample buffer and were boiled for 10 min at 95 °C. A protein standard and the samples were then loaded onto the gel. Electrophoresis was performed at 200 V and 400 mA per gel for 45 min followed by western blot analysis.

A semi-dry western blot was performed to transfer DT-fragments from SDS gels onto polyvinylidene fluoride (PVDF) membranes (Bio-Rad, Feldkirchen, Germany) at 20 V for 30 min. After western blotting, the membrane was blocked with M-PBST for 60 min at room temperature. Detection of DT-fragments was performed indirectly via developed DT-neutralizing human antibodies followed by Fcγ specific AP-conjugated secondary antibody (109-055-098, Jackson Immuno Research Laboratories, Cambridgeshire, UK) in a 1:20,000 dilution in MPBST. After 1 h incubation at room temperature, the membrane was washed with PBST and AP-substrate buffer (100 mM Tris HCl pH 9.5, 0.5 mM MgCl_2_) and bound antibodies were visualized with nitro blue tetrazolium chloride (0.30 mg/mL) and 5-Bromo-4-chloro- 3-indolyl phosphate (0.15 mg/mL) in AP substrate buffer. Finally, the membrane was washed with water and dried. For documentation, the blots were scanned.

### Epitope mapping by phage display

For epitope mapping, single gene libraries were constructed as described previously^64^. In brief, the DT encoding DNA was fragmented by sonication, polished and blunt end cloned into the digested pHORF3 vector^50,82^. For transformation, electro-competent *E. coli* cells SS320 (Lucigen, Middleton, USA) were used. The efficiency of cloning was tested with a colony PCR and the rate of complete fragments were determined. The libraries were further packaged with Hyperphage^45,46^. The produced phage particles were stored at −80 °C after adding glycerine (20% (v/v)).

The panning in solution for epitope mapping was performed as described previously^69^. In brief, 1 µg of an unrelated antibody (negative control) was coated in 100 µL PBS overnight at 4 °C in a cavity of a Costar High Binding microtiter plate. The wells were then blocked with panning block for 1 h at RT and then washed 3x with PBST using a microplate washer. Next, 10^9^ cfu of the DT-fragment-phage library was diluted in 150 µL 2% (w/v) BSA in PBST, added to the negative control well, and incubated for 1 h at room temperature. Protein A coupled magnetic beads (SureBeads, BioRad) were prepared according to manufacturer’s instructions. Preincubated phage was transferred to a protein low binding (PLB) microtube (Sarstedt) and co-incubated with 15 µL Protein A beads for 1 h on a programmable rotator-mixer (PRM) (PTR-30 Grantbio) to further remove sticky or unspecific phage. The magnetic beads were then removed with a magnetic rack and discarded. 100 ng of the DT neutralizing antibody was added to the remaining phage and incubated for 2 h on a rotator-mixer. 15 µL of freshly prepared magnetic protein A beads were added and incubated for another 30 minutes followed by a separation of the beads on a magnetic rack. The supernatant was discarded and the beads were washed 10x with 1 mL PBST and then eluted with 150 µL trypsin (10 µg/mL) and incubated at 37°C for 30 min. The identification of MERs was performed by ELISA. In brief, for the screening ELISA, 100 ng of antibody was coated on 96 well microtitre plates (MaxiSorp, Thermo Fisher Scientific) in PBS pH 7.4 overnight at 4°C. Following coating, the wells were washed with PBST and blocked with 2% MPBST for 1 h at RT, followed by three washing steps with PBST. Supernatant containing DT-fragment bearing phage were mixed with 2% MPBST (1:4) and incubated in the antibody coated plates for 90–120 min at RT followed by three PBST washing cycles. Bound phage was detected using HRP conjugated mouse anti-M13 antibody (27-9421-01, GE Healthcare) diluted 1:40,000 in 2% MPBST and visualized with TMB substrate (20 parts TMB solution A and 1 part TMB solution B). After stopping the reaction by addition of 1 N H_2_SO_4_, absorbance at 450 nm with a 620 nm reference was measured in an ELISA plate reader (Epoch, BioTek). The phagemids encoding the Bound DT-fragments were sequenced and aligned to the DT sequence to identify the MER.

### Microscale thermophoresis for affinity determination

DT was labelled in PBS pH 7.4 using the Amine Reactive Monolith Protein Labeling Kit RED-NHS 2nd Generation (NanoTemper, Munich, Germany) following the manufacturer’s protocol. In total, 5 nM of labelled DT was premixed in a 384 well non-binding polystyrene plate (Corning) with 12 different concentrations (1:2 dilution) of the respective antibody or scFv-Fc in PBS by Precision XS (BioTek Instruments). For Microscale Thermophoresis (MST) measurements with Monolith NT. Automated (NanoTemper) in Monolith NT. Automated Capillary Chips (NanoTemper), an excitation power of 20% and a high MST power of 60% was applied. The Hill coefficient was determined by MO Affinity Analysis software (NanoTemper) for the IgGs after 10 sec of heating and for the scFv-Fc after 15 sec of heating to gain sufficient amplitude signals. All measurements were performed in triplicate resulting in signal to noise ratios over 15.

### *In vivo* efficacy of IgGs by intradermal challenge in guinea pigs

Potency of IgGs by intradermal challenge was performed at NIBSC. *In vivo* studies are regulated by the Animals (Scientific Procedures) Act 1986 in accordance with EC Directive 2010/63/EU. Studies were reviewed and approved by the NIBSC Animal Welfare and Ethical Review Body and performed under a Project Licence granted by the UK Home Office. For the neutralization assays, female guinea pigs (Dunkin Hartley strain) weighing between 350–450 g were obtained from B&K Universal (Marshall BioResources UK) Animals (up to 8 per pen) were housed in rectangular or square floor pens with a metal and perspex construction, above minimum size requirements defined by UK legislation. Animals had olfactory and visual stimulation between the pens with autoclaved dust-free wood shavings as bedding (‘eco-pure’ supplied by Datesand). Husbandry conditions were a 12 h light, 12 h dark cycle, including ‘dawn and dusk’ half-light periods of 30 minutes each. Temperature was maintained between 16–23 degrees Celsius. A pelleted diet (‘FD1’ supplied by LBS), filtered mains water (bottle and bowl) and autoclaved hay were all provided *ad libitum*. Washed fresh greens were provided 3 times weekly. Environmental enrichment including plastic and/or cardboard boxes, tunnels and shelters, and nesting material were also provided in each pen. Animals were health checked at least once per day as stock, and up to 3 times daily whilst on test by trained and competent technical staff, with attention to the size and degree of erythema at the injection sites and the general condition and behaviour of the animals.

The assay was performed according to the method described in the European Pharmacopoeia monograph for Diphtheria Antitoxin^83^. A series of 6 dilutions of antibody sample (either individually or as a combination) were mixed with a fixed volume of DT (EDQM Biological Reference Preparation, batch 1) at a DT dose level of Lr/100. The Lr/100 dose level for this toxin was pre-determined by titration against 0.01 IU/ml of a reference antitoxin (WHO International Standard for Diphtheria Antitoxin Equine, NIBSC code 17/230). A series of 6 dilutions of the reference antitoxin were also prepared and mixed with the same amount of DT in parallel to allow for determination of relative potency. Samples were allowed to stand at RT for 30–40 minutes prior to intradermal injection (0.2 mL) into the shaved flank of a guinea pig. A group of 2 animals was used for each antibody sample (or combination) or the reference antitoxin with 3 dilutions injected per flank. For each sample, the largest volume of antitoxin failing to protect guinea pigs from the local erythematous effect of DT was defined as the end point and potency was determined relative to the end point for the reference antitoxin, after taking into account total dilution factor for the sample.

## Supplementary information

Supplementary Information
