# Supplementary material for: Human antibodies neutralizing diphtheria toxin in vitro and in vivo
Source: Sci Rep. 2020 Jan 17;10:571. doi: 10.1038/s41598-019-57103-5 (PMC6969050; doi:10.1038/s41598-019-57103-5)
Supplement: Supplementary file 1 — Supplementary Information [file 41598_2019_57103_MOESM1_ESM.zip › Supplementary figures revised.docx]

**Supplementary figures**

**Human antibodies neutralizing diphtheria toxin in vitro and in vivo**

Esther Veronika Wenzel^1^, Margarita Bosnak^1^, Robert Tierney^2^, Maren Schubert^1^, Jeffrey Brown^3^, Stefan Dübel^1^, Androulla Efstratiou^4^, Dorothea Sesardic^2^, Paul Stickings^†,2^ and Michael Hust^†,1*^

^1^ Technische Universität Braunschweig, Institute for Biochemistry, Biotechnology and Bioinformatics, Department of Biotechnology, Braunschweig, Germany

^2^ National Institute for Biological Standards and Control (NIBSC), Division of Bacteriology, Potters Bar, United Kingdom

^3^ PETA International Science Consortium Ltd, London, United Kingdom

^4^ WHO Collaborating Centre for Diphtheria and Streptococcal Infections, London, UK

† both senior authors contributed equally

* corresponding author:

Michael Hust

Technische Universität Braunschweig

Institute for Biochemistry, Biotechnology and Bioinformatics,

Department of Biotechnology,

Spielmannstr. 7

38106 Braunschweig, Germany

m.hust@tu-bs.de

**Supplementary figures legends**

Supplementary figure 1: Analysis of the DT gene coverage. 74 clones of both DT gene libraries were analyzed by sequencing the pHORF3 vector.

Supplementary figure 2: Identification of the minimal epitope region (MER) of 19 neutralizing antibodies. A) antibody ewe191-A7 B) ewe191-C11 C) ewe191-D1 D) ewe191-H10 E) ewe192-D7 F) ewe192-D8 G) ewe192-G12 H) ewe192-H7 I) ewe192-H8 J) ewe371-B12 K) ewe372-A8 L) ewe372-C4 M) ewe372-C5 N) ewe372-D11 O) ewe372-D7 P) ewe372-H1-S Q) ewe372-H1-W R) ewe375-D4 S) ewe375-H4 and T) ewe191-F11 as example for inconclusive results.

Supplementary figure 3: Microscale Thermophorese measurement of six neutralizing antibodies in scFv-Fc and IgG format. All measurements were performed in triplicates and signals was measured for scFv-Fc after 15 sec and for IgG format after 10 sec. Data was analyzed with MO Affinity Analysis software (Nanotemper, Munich, Germany).
