# Supplementary figures and images for: Human antibodies neutralizing diphtheria toxin in vitro and in vivo
Source: Sci Rep. 2020 Jan 17;10:571. doi: 10.1038/s41598-019-57103-5 (PMC6969050; doi:10.1038/s41598-019-57103-5)

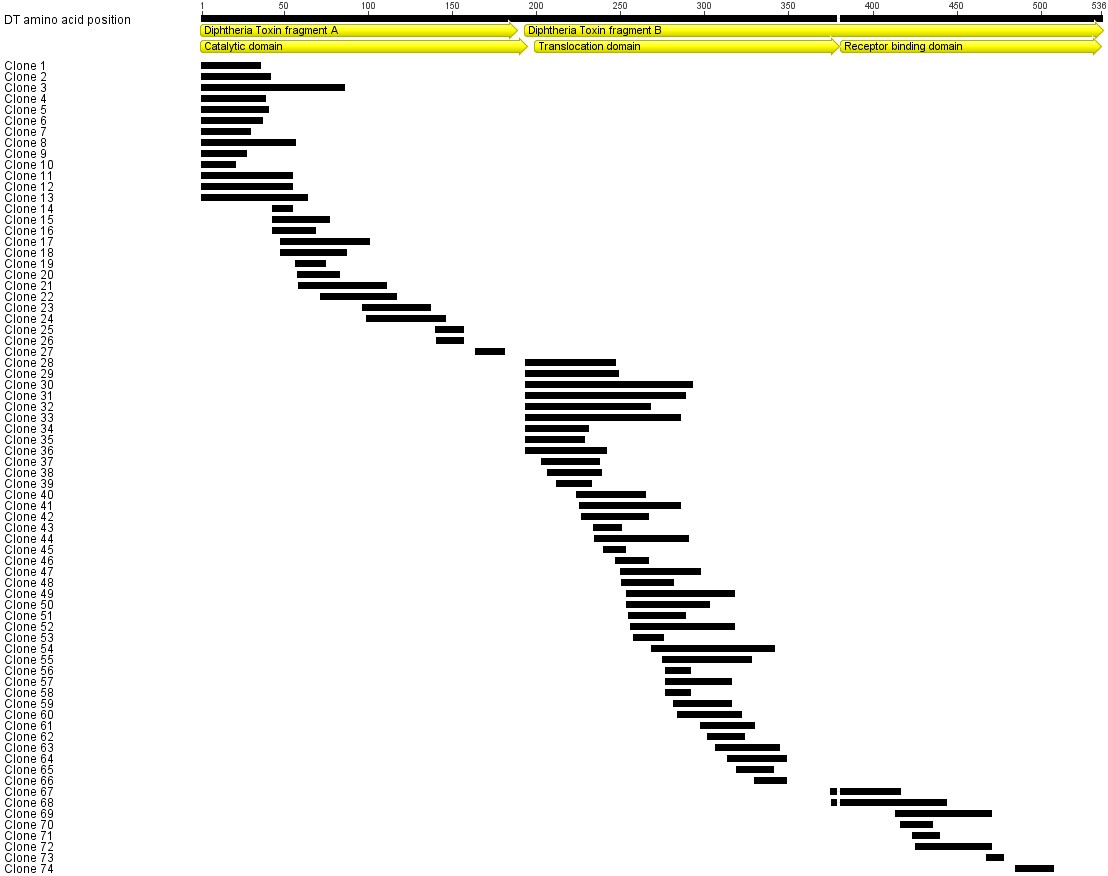

Supplement: Supplementary file 1 — Supplementary Information [file 41598_2019_57103_MOESM1_ESM.zip › Supplementary figure 1.jpg]

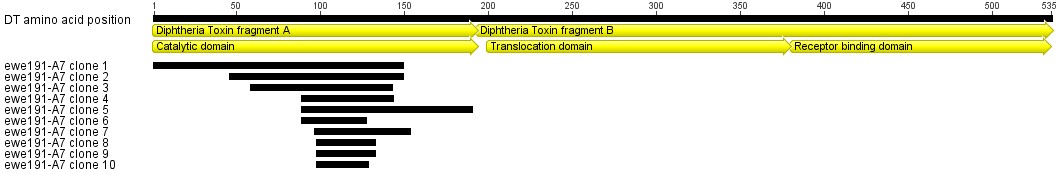

Supplement: Supplementary file 1 — Supplementary Information [file 41598_2019_57103_MOESM1_ESM.zip › Supplementary figure 2A.jpg]

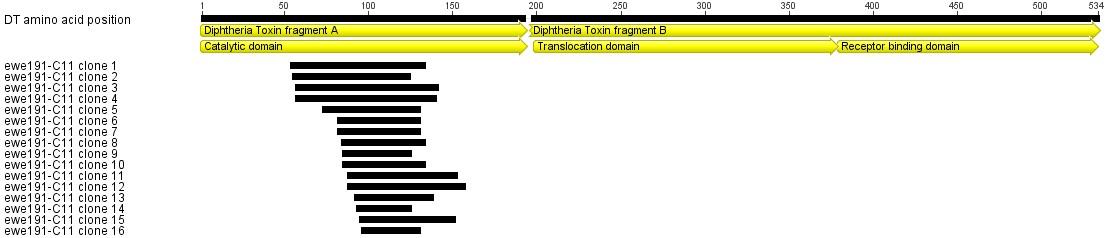

Supplement: Supplementary file 1 — Supplementary Information [file 41598_2019_57103_MOESM1_ESM.zip › Supplementary figure 2B.jpg]

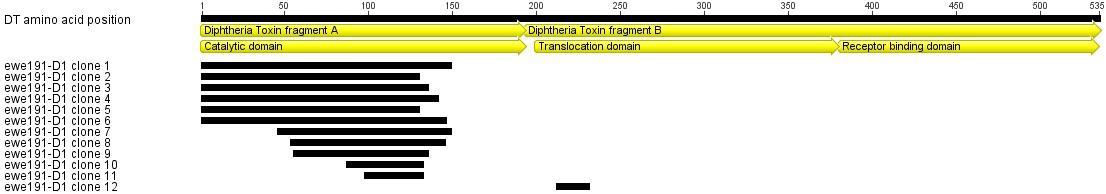

Supplement: Supplementary file 1 — Supplementary Information [file 41598_2019_57103_MOESM1_ESM.zip › Supplementary figure 2C.jpg]

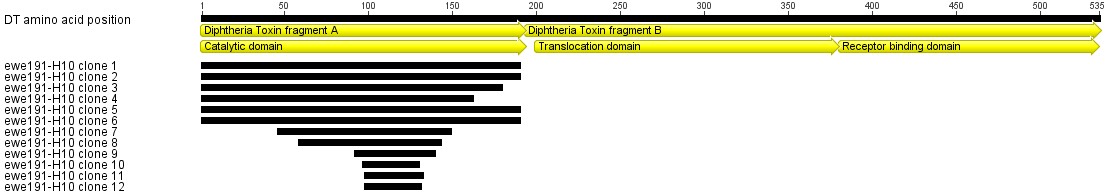

Supplement: Supplementary file 1 — Supplementary Information [file 41598_2019_57103_MOESM1_ESM.zip › Supplementary figure 2D.jpg]

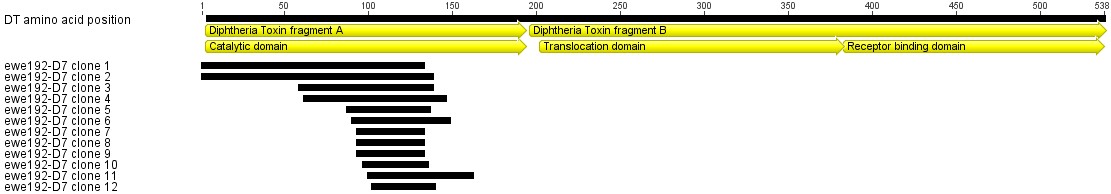

Supplement: Supplementary file 1 — Supplementary Information [file 41598_2019_57103_MOESM1_ESM.zip › Supplementary figure 2E.jpg]

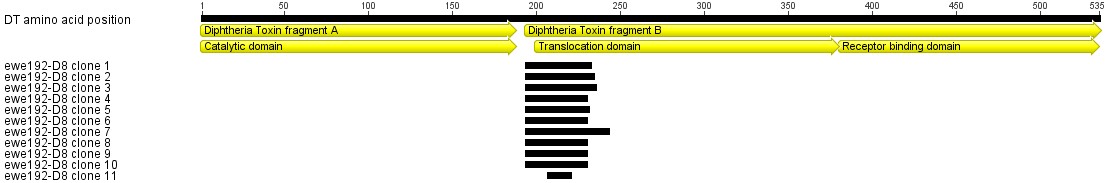

Supplement: Supplementary file 1 — Supplementary Information [file 41598_2019_57103_MOESM1_ESM.zip › Supplementary figure 2F.jpg]

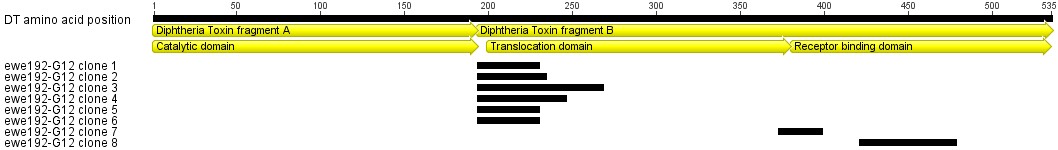

Supplement: Supplementary file 1 — Supplementary Information [file 41598_2019_57103_MOESM1_ESM.zip › Supplementary figure 2G.jpg]

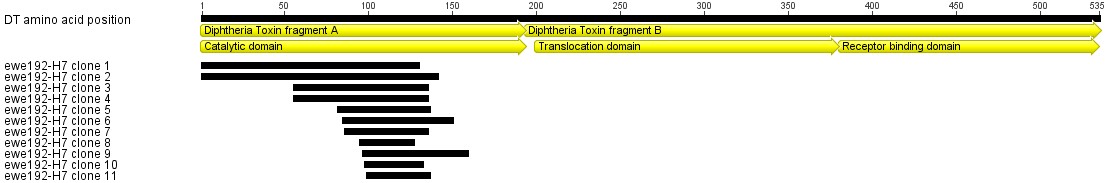

Supplement: Supplementary file 1 — Supplementary Information [file 41598_2019_57103_MOESM1_ESM.zip › Supplementary figure 2H.jpg]

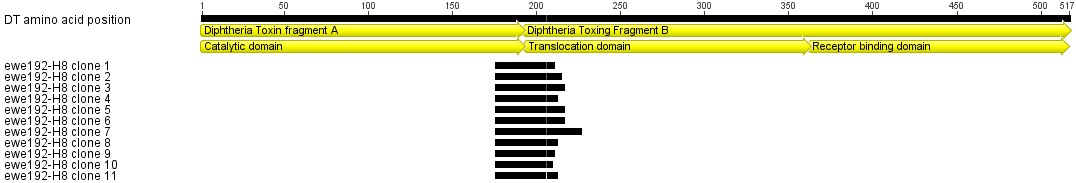

Supplement: Supplementary file 1 — Supplementary Information [file 41598_2019_57103_MOESM1_ESM.zip › Supplementary figure 2I.jpg]

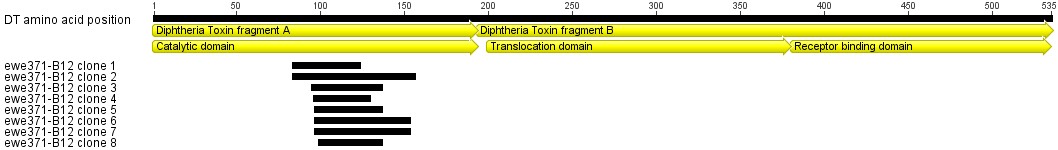

Supplement: Supplementary file 1 — Supplementary Information [file 41598_2019_57103_MOESM1_ESM.zip › Supplementary figure 2J.jpg]

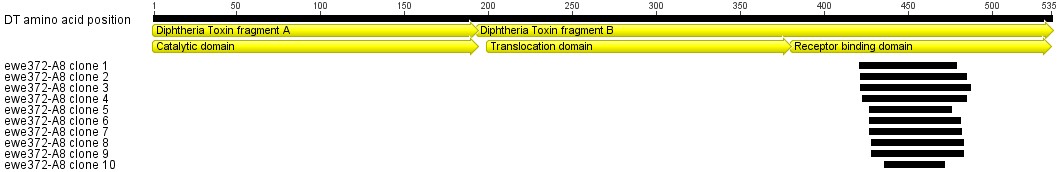

Supplement: Supplementary file 1 — Supplementary Information [file 41598_2019_57103_MOESM1_ESM.zip › Supplementary figure 2K.jpg]

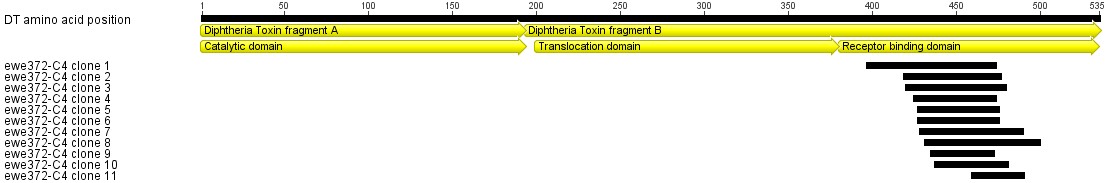

Supplement: Supplementary file 1 — Supplementary Information [file 41598_2019_57103_MOESM1_ESM.zip › Supplementary figure 2L.jpg]

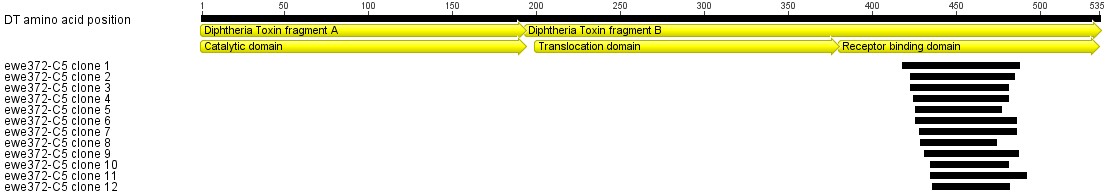

Supplement: Supplementary file 1 — Supplementary Information [file 41598_2019_57103_MOESM1_ESM.zip › Supplementary figure 2M.jpg]

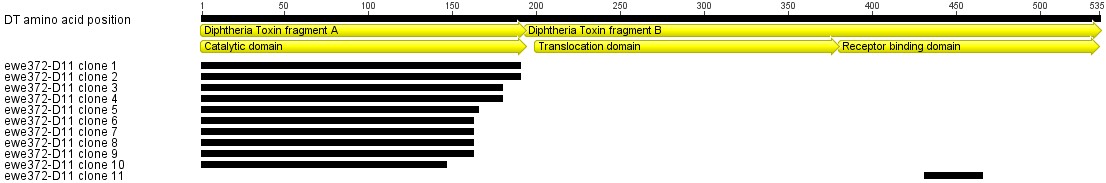

Supplement: Supplementary file 1 — Supplementary Information [file 41598_2019_57103_MOESM1_ESM.zip › Supplementary figure 2N.jpg]

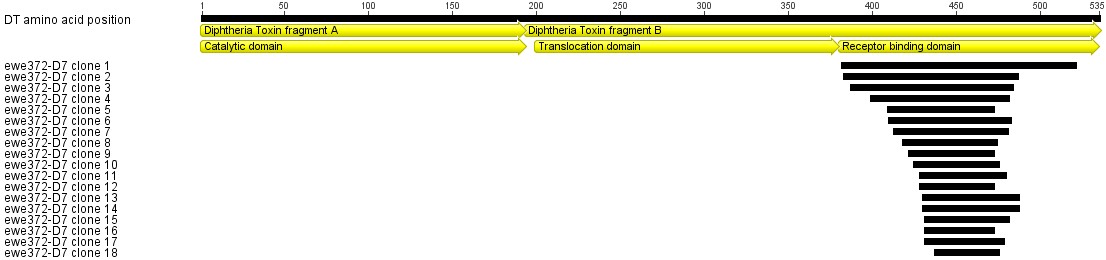

Supplement: Supplementary file 1 — Supplementary Information [file 41598_2019_57103_MOESM1_ESM.zip › Supplementary figure 2O.jpg]

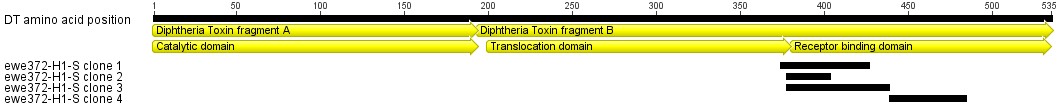

Supplement: Supplementary file 1 — Supplementary Information [file 41598_2019_57103_MOESM1_ESM.zip › Supplementary figure 2P.jpg]

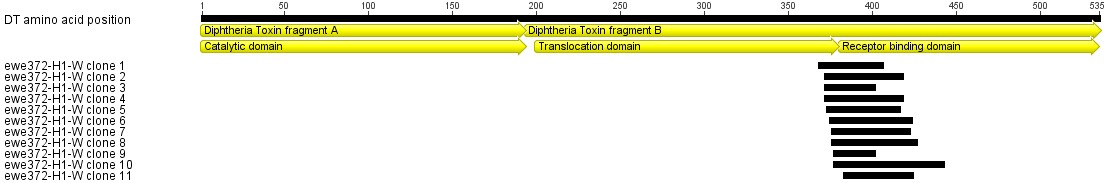

Supplement: Supplementary file 1 — Supplementary Information [file 41598_2019_57103_MOESM1_ESM.zip › Supplementary figure 2Q.jpg]

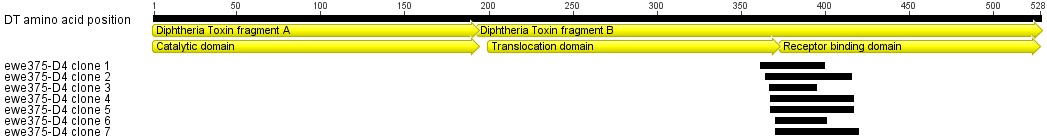

Supplement: Supplementary file 1 — Supplementary Information [file 41598_2019_57103_MOESM1_ESM.zip › Supplementary figure 2R.jpg]

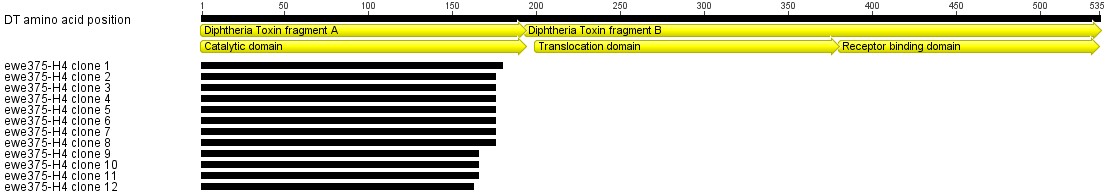

Supplement: Supplementary file 1 — Supplementary Information [file 41598_2019_57103_MOESM1_ESM.zip › Supplementary figure 2S.jpg]

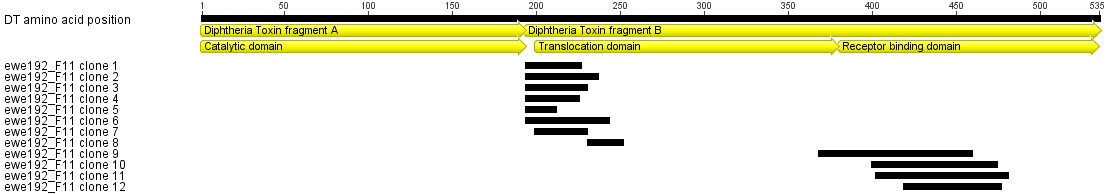

Supplement: Supplementary file 1 — Supplementary Information [file 41598_2019_57103_MOESM1_ESM.zip › Supplementary figure 2T.jpg]

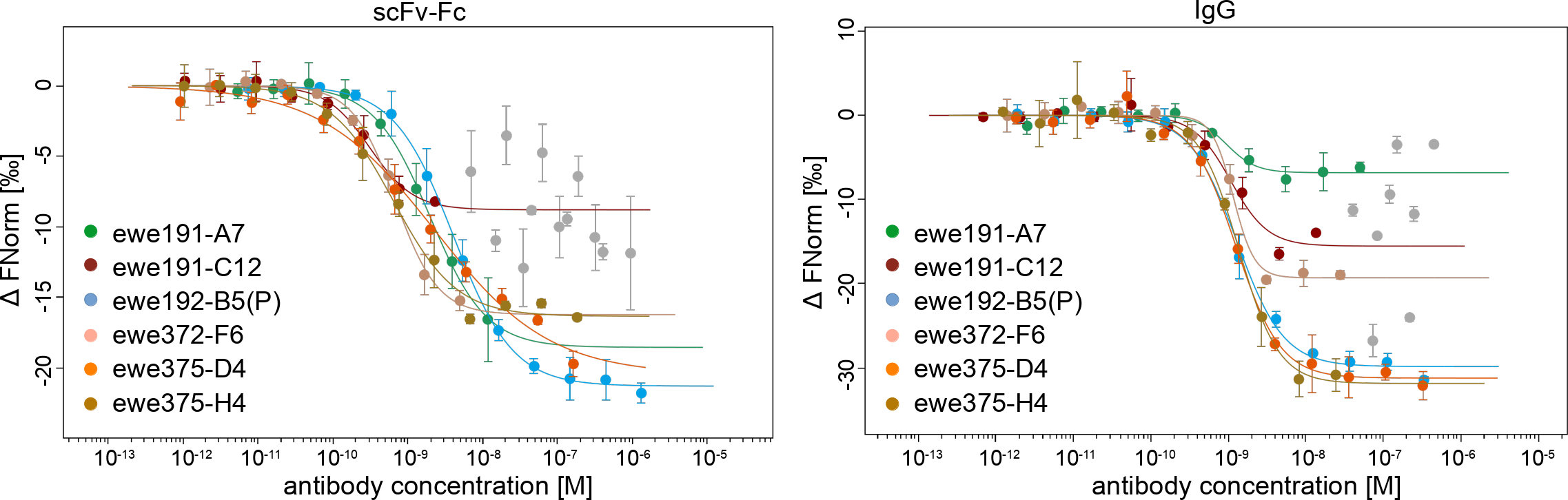

Supplement: Supplementary file 1 — Supplementary Information [file 41598_2019_57103_MOESM1_ESM.zip › Supplementary figure 3.png]
